# A Genome-Wide mQTL Analysis in Human Adipose Tissue Identifies Genetic Variants Associated with DNA Methylation, Gene Expression and Metabolic Traits

**DOI:** 10.1371/journal.pone.0157776

**Published:** 2016-06-20

**Authors:** Petr Volkov, Anders H. Olsson, Linn Gillberg, Sine W. Jørgensen, Charlotte Brøns, Karl-Fredrik Eriksson, Leif Groop, Per-Anders Jansson, Emma Nilsson, Tina Rönn, Allan Vaag, Charlotte Ling

**Affiliations:** 1 Department of Clinical Sciences, Epigenetics and Diabetes, Lund University Diabetes Centre, Clinical Research Centre, Malmö, Sweden; 2 Department of Endocrinology, Diabetes and Metabolism, Rigshospitalet, Copenhagen, Denmark; 3 Department of Clinical Sciences, Vascular Diseases, Lund University, Malmö, Sweden; 4 Department of Clinical Sciences, Diabetes and Endocrinology, Lund University Diabetes Centre, Clinical Research Centre, Malmö, Sweden; 5 The Lundberg Laboratory for Diabetes Research, Center of Excellence for Cardiovascular and Metabolic Research, Department of Molecular and Clinical Medicine, Institute of Medicine, Sahlgrenska University Hospital, University of Gothenburg, Gothenburg, Sweden; Medical University Hamburg, University Heart Center, GERMANY

## Abstract

Little is known about the extent to which interactions between genetics and epigenetics may affect the risk of complex metabolic diseases and/or their intermediary phenotypes. We performed a genome-wide DNA methylation quantitative trait locus (mQTL) analysis in human adipose tissue of 119 men, where 592,794 single nucleotide polymorphisms (SNPs) were related to DNA methylation of 477,891 CpG sites, covering 99% of RefSeq genes. SNPs in significant mQTLs were further related to gene expression in adipose tissue and obesity related traits. We found 101,911 SNP-CpG pairs (mQTLs) in *cis* and 5,342 SNP-CpG pairs in *trans* showing significant associations between genotype and DNA methylation in adipose tissue after correction for multiple testing, where *cis* is defined as distance less than 500 kb between a SNP and CpG site. These mQTLs include reported obesity, lipid and type 2 diabetes loci, e.g. *ADCY3/POMC*, *APOA5*, *CETP*, *FADS2*, *GCKR*, *SORT1* and *LEPR*. Significant mQTLs were overrepresented in intergenic regions meanwhile underrepresented in promoter regions and CpG islands. We further identified 635 SNPs in significant *cis*-mQTLs associated with expression of 86 genes in adipose tissue including *CHRNA5*, *G6PC2*, *GPX7*, *RPL27A*, *THNSL2* and *ZFP57*. SNPs in significant mQTLs were also associated with body mass index (BMI), lipid traits and glucose and insulin levels in our study cohort and public available consortia data. Importantly, the Causal Inference Test (CIT) demonstrates how genetic variants mediate their effects on metabolic traits (e.g. BMI, cholesterol, high-density lipoprotein (HDL), hemoglobin A1c (HbA1c) and homeostatic model assessment of insulin resistance (HOMA-IR)) via altered DNA methylation in human adipose tissue. This study identifies genome-wide interactions between genetic and epigenetic variation in both *cis* and *trans* positions influencing gene expression in adipose tissue and *in vivo* (dys)metabolic traits associated with the development of obesity and diabetes.

## Introduction

Genetic factors contribute to the risk of complex metabolic diseases such as obesity and type 2 diabetes. Although genome-wide association studies (GWAS) have identified numerous genetic loci influencing the risk of developing obesity and type 2 diabetes, only a few of these loci have been linked to the molecular mechanisms contributing to the phenotype outcome [1]. Moreover, the identified genetic loci do only explain a modest proportion of the estimated heritability of these diseases and additional genetic mechanisms remain to be found. These may include genetic variants interacting with epigenetic modifications.

The phenomenon of epigenetic modifications are of interest to study for their possible involvement in phenotype transmission and predisposition to complex human diseases, including obesity and type 2 diabetes [2,3]. Epigenetics has been defined as heritable changes in gene function that occur without alterations in the DNA sequence and includes the molecular mechanism of DNA methylation [4]. In differentiated mammalian cells, DNA methylation occurs primarily at cytosines in CG dinucleotides, so called CpG methylation, which is associated with regulation of cell specific gene expression [5,6]. DNA methylation patterns are mainly established early in life, but may also be dynamic and change in response to environmental stimulations such as diet and exercise [7–10]. Concurrently, once epigenetic modifications are introduced they can be stable and inherited [11,12], making epigenetics a potentially important pathogenic mechanism in complex metabolic diseases. Interestingly, twin studies provide evidence for an underlying genetic effect on DNA methylation patterns [13–16]. For example using monozygotic and dizygotic twins, Grundberg et al showed that as much as 37% of the methylation variance can be attributed to genetic factors, which is in line with previous studies [15,16]. In addition, recent studies showed that common genetic variation regulates DNA methylation levels, so called methylation quantitative trait loci (mQTLs) [16–20]. However, most of these studies have been limited to analyses of ~0.1% of human CpG sites in promoter regions [17–19] or restricted to SNPs located within 100 kb from analyzed CpG sites [16]. It remains to be tested if genetic and epigenetic variation interacts throughout the genome in human adipose tissue and subsequently affect gene expression and metabolic traits such as BMI, lipid levels and hemoglobin A1c (HbA1c) in the studied individuals.

The aim of the present study was therefore to perform a genome-wide mQTL analysis in human adipose tissue, investigating both *cis* and *trans* effects of genetic variation on DNA methylation covering most genes and regions in the human genome. Identified mQTLs were followed-up and related to gene expression in adipose tissue. Additionally, since the adipose tissue contributes to whole body energy homeostasis by glucose uptake, triglyceride storage and adipokine secretion, we investigated if the identified SNPs in significant mQTLs affect metabolic traits that are associated with increased risk of obesity and type 2 diabetes in the studied cohort. We further used a causal inference test (CIT) [21] to model the potential causal relationships between genotype, DNA methylation and metabolic phenotypes.

The present study provides the first detailed map of genetic loci in both *cis* and *trans* positions affecting the genome-wide DNA methylation pattern in human adipose tissue as well as numerous metabolic traits. Identified mQTLs cover known lipid, obesity and diabetes loci. Our study highlights that interaction analysis between genetic and epigenetic variation in a tissue of relevance for metabolic diseases may give new insights to biological processes affecting disease susceptibility.

## Results

### Associations between genetic variation and DNA methylation in human adipose tissue–a genome-wide mQTL analysis

**Fig 1:**
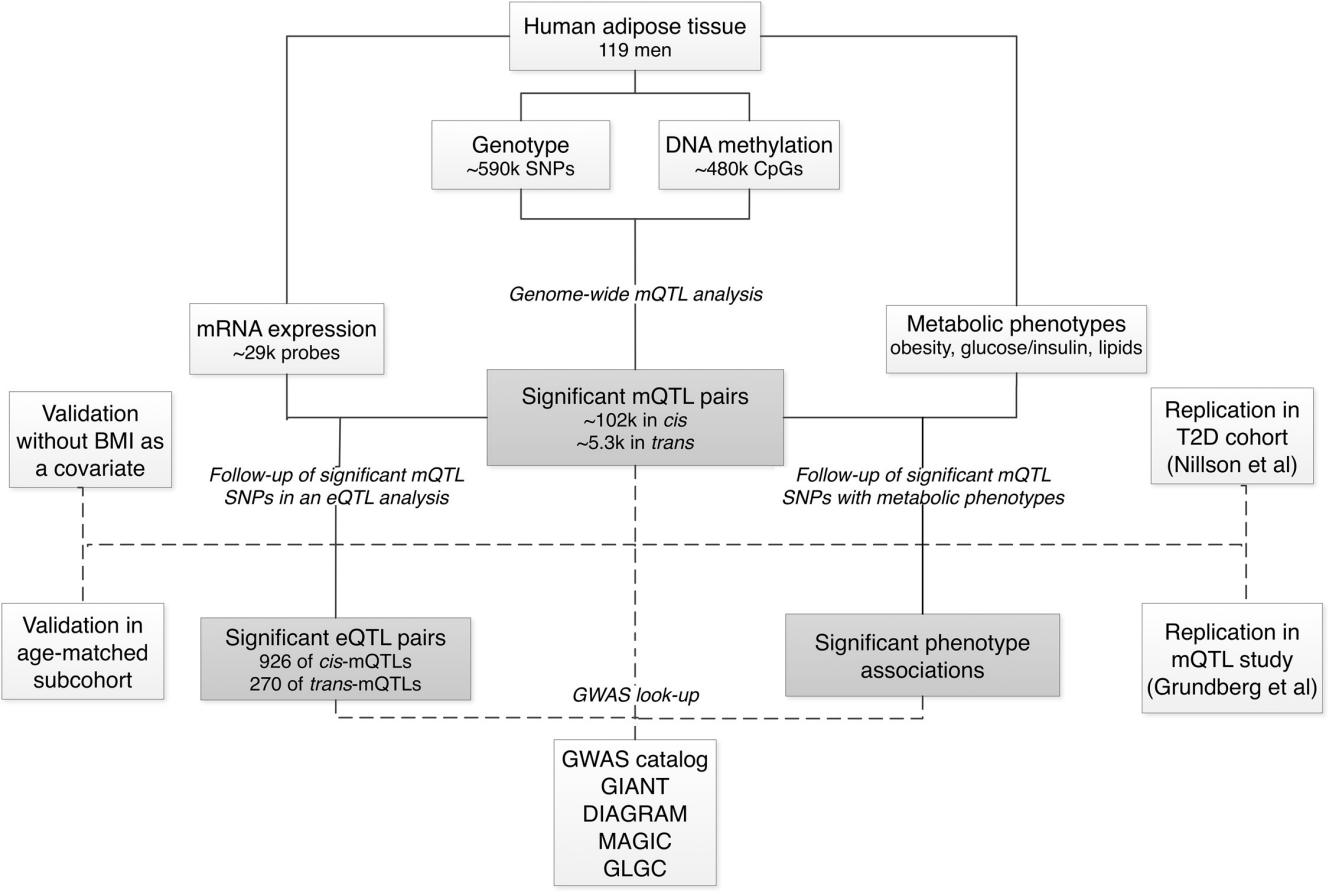
Analysis flowchart of the study.

To examine and map underlying genetic control of DNA methylation patterns in human adipose tissue, we performed a genome-wide mQTL analysis (**Fig 1**). While most previous mQTL studies have been limited to analysis of ~0.1% of human CpG sites [17–19] or SNPs within 100 kb from analyzed CpG sites [16] we performed the first combined *cis*- and *trans*-mQTL analysis covering DNA methylation of most genes and genomic regions in human adipose tissue of 119 Scandinavian men (**Table 1**). Here, we pairwise associated genotype data of 592,794 common SNPs (MAF>0.05) with DNA methylation of 477,891 CpG sites throughout the human genome using a linear regression model including sub-cohort, age and BMI as covariates.

**Table 1:** Characteristics of 119 Scandinavian men included in the mQTL analysis.

Phenotype	Mean ± SD	Min	1^st^. quartile	Median	3^rd^ quartile	Max
Age (years)	31.03 ± 12.3	22	24	25	35	80
Fasting Glucose (mmol/l)	4.76 ± 0.64	3.2	4.4	4.7	5	7
Fasting Insulin (pmol/l)	37.29 ± 22.43	8	23.1	33	43.3	181.3
Weight (kg)	80.86 ± 11.6	57.2	72.6	80.4	89.57	112.7
BMI (kg/m^2^)	24.91 ± 3.7	16.4	22.2	24.6	27.15	39
Waist (cm)	90.31 ± 11.5	68	80.75	91	98.25	129
Hip (cm)	97.55 ± 8.8	78	91.75	98	104.2	113
Waist-Hip ratio	0.9 ± 0.06	0.79	0.87	0.9	0.92	1
Cholesterol (mmol/l)	4.5 ± 0.84	2.1	3.9	4.5	5.1	7.1
Triglycerides (mmol/l)	1.14 ± 0.66	0.3	0.72	1	1.3	4.9
HDL (mmol/l)	1.16 ± 0.2	0.5	1	1.13	1.37	1.86
LDL (mmol/l)	2.8 ± 0.77	1	2.3	2.8	3.5	4.7
HbA1c (%)	4.93 ± 0.48	3.7	4.7	5	5.2	6.4
HOMA-IR	1.15 ± 0.78	0.2	0.7	1	1.4	6.5
HOMA-B	133.69 ± 226.33	19.2	56	75.6	118.9	1834

**Fig 2:**
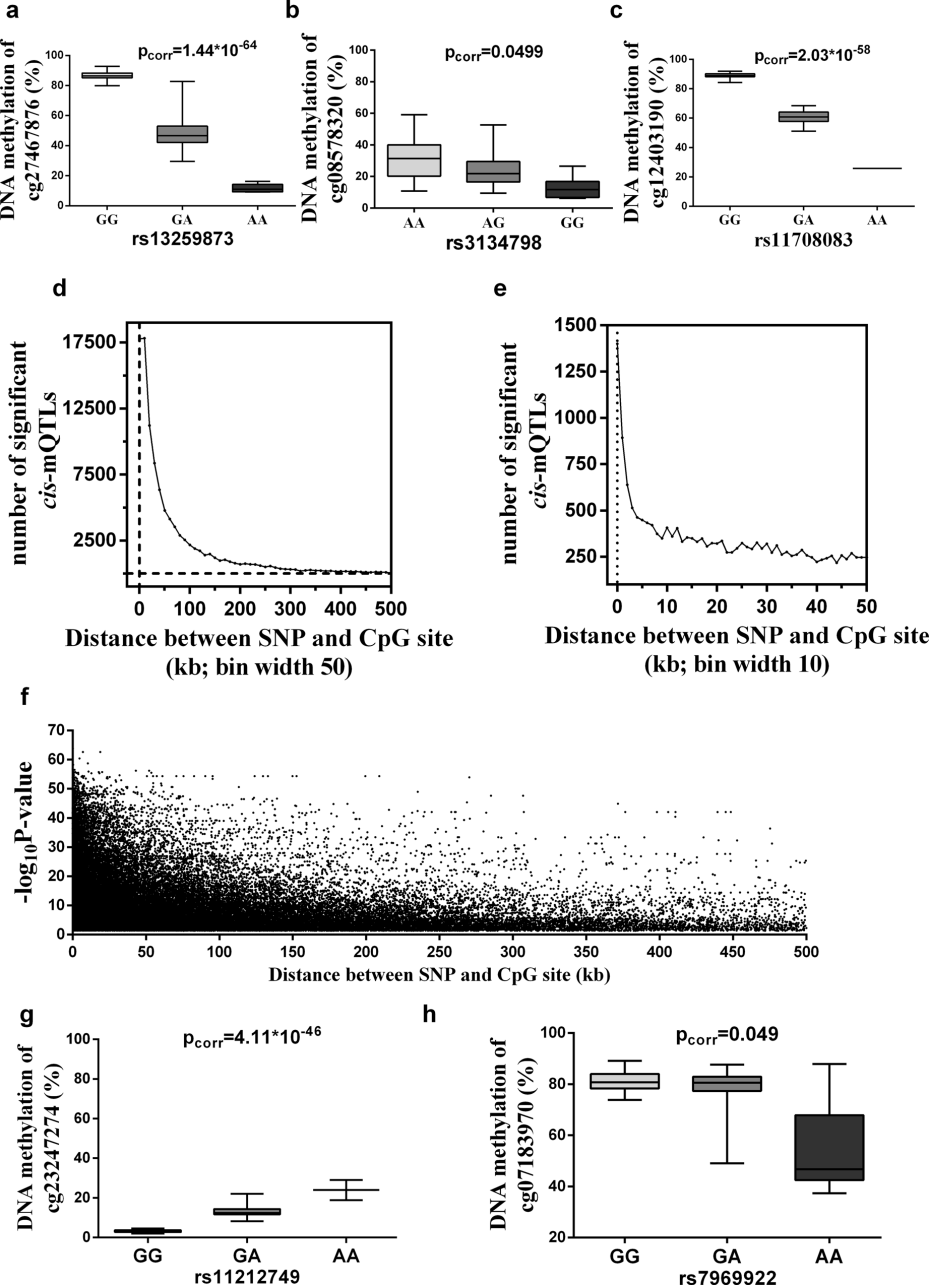
Associations between SNPs and DNA methylation in human adipose tissue. A genome-wide mQTL analysis in human adipose tissue was performed by associating SNPs with DNA methylation of CpG sites located in either *cis* (≤500 kb) or *trans*. Boxplots of (**a**) the top *cis*-mQTL, (**b**) the bottom *cis*-mQTL, and (**c**) the top *cis*-mQTL where the SNP introduces or removes a CpG site (CpG-SNP), showing significant associations between SNPs (*genotype groups*, *x-axis*) and DNA methylation of CpG sites (*%*, *y-axis*). (**d-e**) The frequency of associations (*y-axis*) is plotted in relation to the relative distance between SNPs and CpG sites (*kb*, *x-axis*) of significant *cis*-mQTLs. In (**d**) the full *cis*-mQTL distance of 500 kb is represented and the frequency of significant *cis*-mQTLs within each distance bin of 10kb are plotted, and, in (**e**) the region of 0-50kb is zoomed and the frequency of significant *cis*-mQTLs within in each distance bin of 1kb is plotted. (**f**) Histogram showing the strength of association (*-log*_*10*_ *p-value*, *y-axis*) in relation to distance between SNP and CpG site (*kb*, *x-axis*) of significant *cis*-mQTLs. The most frequent and strongest association signals of *cis*-mQTLs are shown within SNPs located close to CpG sites. (**g-h**) Boxplots of (**g**) the top *trans*-mQTL, and (**h**) the bottom *trans*-mQTL, showing significant associations between SNPs (*genotype groups*, *x-axis*) and DNA methylation of CpG sites (*%*, *y-axis*). p_corr_, p-values have been corrected for multiple testing by a modified Bonferroni correction where the LD structure of SNPs is taken into account (see methods).

The *cis*-mQTL analysis was limited to SNPs located within 500 kb of either side of the analyzed CpG sites. Here, we detected 101,911 SNP-CpG pairs (mQTLs) showing significant associations between genotype and the degree of DNA methylation after correction for multiple testing (**see** Methods), corresponding to 51,143 unique SNPs and 15,208 unique CpG sites (**Table 2**and **S1 Table**). Of these 15,208 significant CpG sites, 10,064 were annotated to 5,589 unique genes (**Table 2)** and 5,144 CpG sites were annotated to intergenic regions. The most and least significant *cis*-mQTLs are shown in **Fig 2A–2B.**

**Table 2:** Number of significant mQTL results in human adipose tissue.

	*cis-*mQTL	*trans*-mQTL
SNP-CpG pairs	101,911	5,342
SNPs	51,143	2,735
CpG sites	15,208	596
Unique genes	5,589	375

Significance threshold < 0.05 after Bonferroni correction for multiple testing.

Correction value *cis* = 104,023,091

Correction value *trans* = 211,781,637,483.

Previously, we reported that approximately 50% of type 2 diabetes associated SNPs identified by GWAS either introduce or remove a CpG site, a so called CpG-SNP. These CpG-SNPs were further associated with differential DNA methylation of the CpG-SNP site in human pancreatic islets [22]. Among the significant *cis*-mQTLs in the present study, 447 SNPs were located within a CpG site, i.e. the distance between a SNP and CpG site is 0 or 1 and thereby remove or introduce a CpG site–CpG-SNPs (**S1 Table**). The most significant mQTL among these 447 *cis*-mQTLs is presented in **Fig 2C.**

When a distance analysis was performed, we found an overrepresentation (p < 2.2^−16^) of SNPs in significant *cis*-mQTL located close to the CpG site (**Fig 2D–2E**), with a median distance between SNPs and CpG sites of significant *cis*-mQTLs of 29.6 kb. Moreover, the strongest association signals were found for SNPs located close to a CpG site (**Fig 2F**).

In the *trans*-mQTL analysis, including SNPs located more than 500 kb from the analyzed CpG sites, we identified 5,342 SNP-CpG pairs showing significant associations between genotypes and the degree of DNA methylation in adipose tissue after correction for multiple testing (**see** Methods), corresponding to 2,735 unique SNPs and 596 unique CpG sites (**Table 2**and **S2 Table**). Among unique CpG sites of significant *trans*-mQTLs, 366 CpG sites were annotated to 375 unique genes (**Table 2**and **S2 Table**) and 230 CpG sites were annotated to intergenic regions. The most and least significant *trans*-mQTLs are shown in **Fig 2G–2H.**

### Genomic distribution of significant mQTLs in human adipose tissue

**Fig 3:**
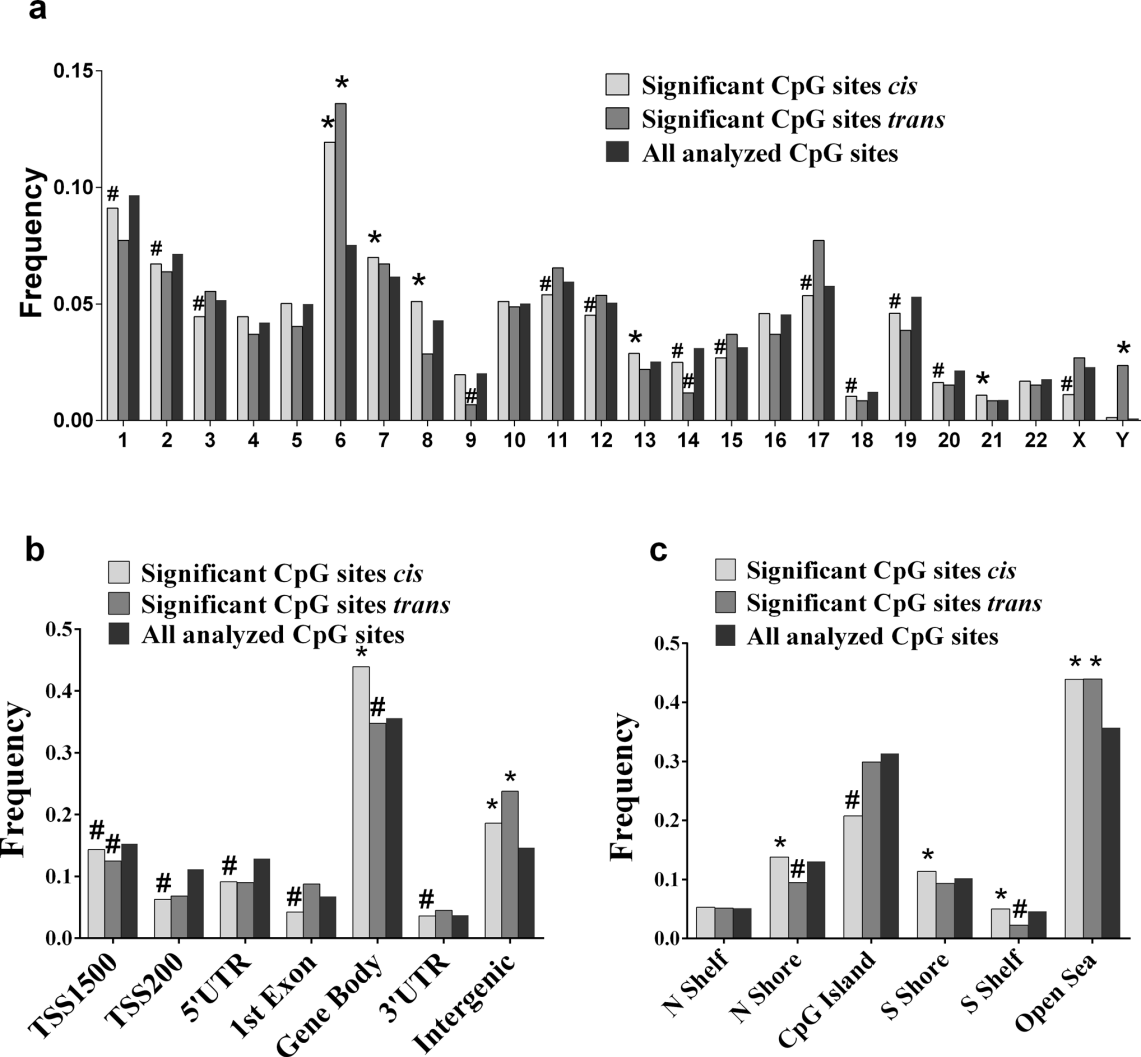
Distribution of CpG sites of significant mQTLs in relation to genomic regions. We examined the chromosomal and genomic distribution of CpG sites in significant mQTLs in human adipose tissue. By using chi-squared-tests, we determined whether the observed frequency of significant CpGs in *cis*- or *trans*-mQTLs differs from the frequency of all analyzed CpG sites for a particular genomic region. The histograms show the distributions of CpGs in relation to (**a**) chromosomes, (**b**) nearest gene, and (**c**) CpG islands. *Frequencies, significantly different (over-represented) from what expected by chance. ^#^Frequencies, significantly different (under-represented) from what expected by chance. Genomic region in relation to nearest gene includes: *TSS 1500* and *TSS 200* (sites located 1500–201 or 200–0 bases upstream of the transcription start site (TSS) respectively), *5’UTR*, *1*^*st*^ *exon*, *gene body*, *3’UTR* and *intergenic region* (not mapped to any of the other regions). Genomic region in relation to CpG island includes: *CpG island*, *shore* (flanking region of CpG island, 0–2000 bp), *shelf* (flanking region of shore, 2000–4000 bp distant from CpG island) and *open sea* (not mapped to any of the other regions).

DNA methylation in proximal promoter and/or enhancer regions is generally thought to have silencing effects on gene transcription, meanwhile DNA methylation in the gene body might stimulate transcriptional elongation and contribute to alternative splicing events [6]. Giving the various functions of DNA methylation in the context of genomic regions, it is of interest to study the underlying mechanisms regulating DNA methylation patterns in different genomic regions. We therefore studied the chromosomal and genomic distribution of CpG sites in significant mQTLs in human adipose tissue. To determine whether the genomic distribution of CpG sites in significant mQTLs differ significantly from all analyzed CpG sites on the array, we performed chi-squared tests. The chromosomal distribution of CpG sites in significant *cis-* and *trans-*mQTLs is shown in **Fig 3A.** We found an overrepresentation of CpG sites in significant *cis*-mQTLs on chromosome 6, 7, 8, 13 and 21 together with an underrepresentation on chromosomes 1, 2, 3, 11, 12, 14, 15, 17, 18, 19, 20 and X when compared to the chromosomal distribution of all analyzed CpG sites (**Fig 3A**). The highest deviation from expectation of CpGs in significant *cis*-mQTLs was observed on chromosome 6 (p-value = 3.4x10^-89^), where the highly polymorphic HLA region is located, a genomic region linked to numerous autoimmune diseases [23,24]. CpG sites in significant *trans*-mQTLs were overrepresented on chromosomes 6 and Y while underrepresented on chromosomes 9 and 14 (**Fig 3A**).

Furthermore, the Infinium HumanMethylation450 BeadChip estimates DNA methylation in several genomic features and the analyzed CpG sites have been annotated based on their genomic location in relation to the nearest gene including genomic regions TSS1500 and TSS200 (1500–201 and 200–0 bases upstream of transcription start site (TSS), respectively), 5’UTR (untraslated region), 1^st^ exon, gene body, 3’UTR and intergenic regions [25]. In the present study, CpG sites in significant *cis*-mQTLs were overrepresented in the intergenic regions and gene body, while significantly underrepresented in the TSS1500, TSS200, 5’UTR, 1^st^ exon and 3'UTR (**Fig 3B**). Among significant *trans*-mQTLs, we found an overrepresentation of CpGs in the intergenic region and underrepresentation in TSS1500 and gene body (**Fig 3B**).

The analyzed CpG sites have also been annotated based on their relation to CpG islands, including the following regions: CpG islands, northern and southern shores, northern and southern shelves and open sea [25]. For CpG sites in significant *cis*-mQTLs, we found an overrepresentation in the open sea, northern- and southern shores as well as in southern shelf (**Fig 3C**). Moreover, an underrepresentation was found in CpG islands (**Fig 3C**). CpGs in significant *trans*-mQTLs showed overrepresentation in the open sea and underrepresentation in northern shore as well as southern shelf (**Fig 3C**).

Next, we performed a KEGG (Kyoto Encyclopaedia of Genes and Genomes) pathway analysis to identify cellular components or biological pathways which show enrichment among genes identified in *cis-* and *trans-* mQTL analyses in human adipose tissue. Using WebGestalt [26], we identified 172 significant (FDR < 0.05) KEGG pathways enriched among 5,589 genes annotated to significant *cis* mQTLs (**S3 Table**), including Metabolic pathways (P_adj_ = 6.3x10^-15^) and Pathways in Cancer (P_adj_ = 7.3x10^-42^) were found among the most enriched KEGG pathways (**S3 Table**). Moreover, 25 KEGG pathways were enriched among 375 genes annotated to significant *trans* mQTLs (**S3 Table**).

### Candidate loci for obesity and diabetes related traits are detected among mQTLs in human adipose tissue

**Fig 4:**
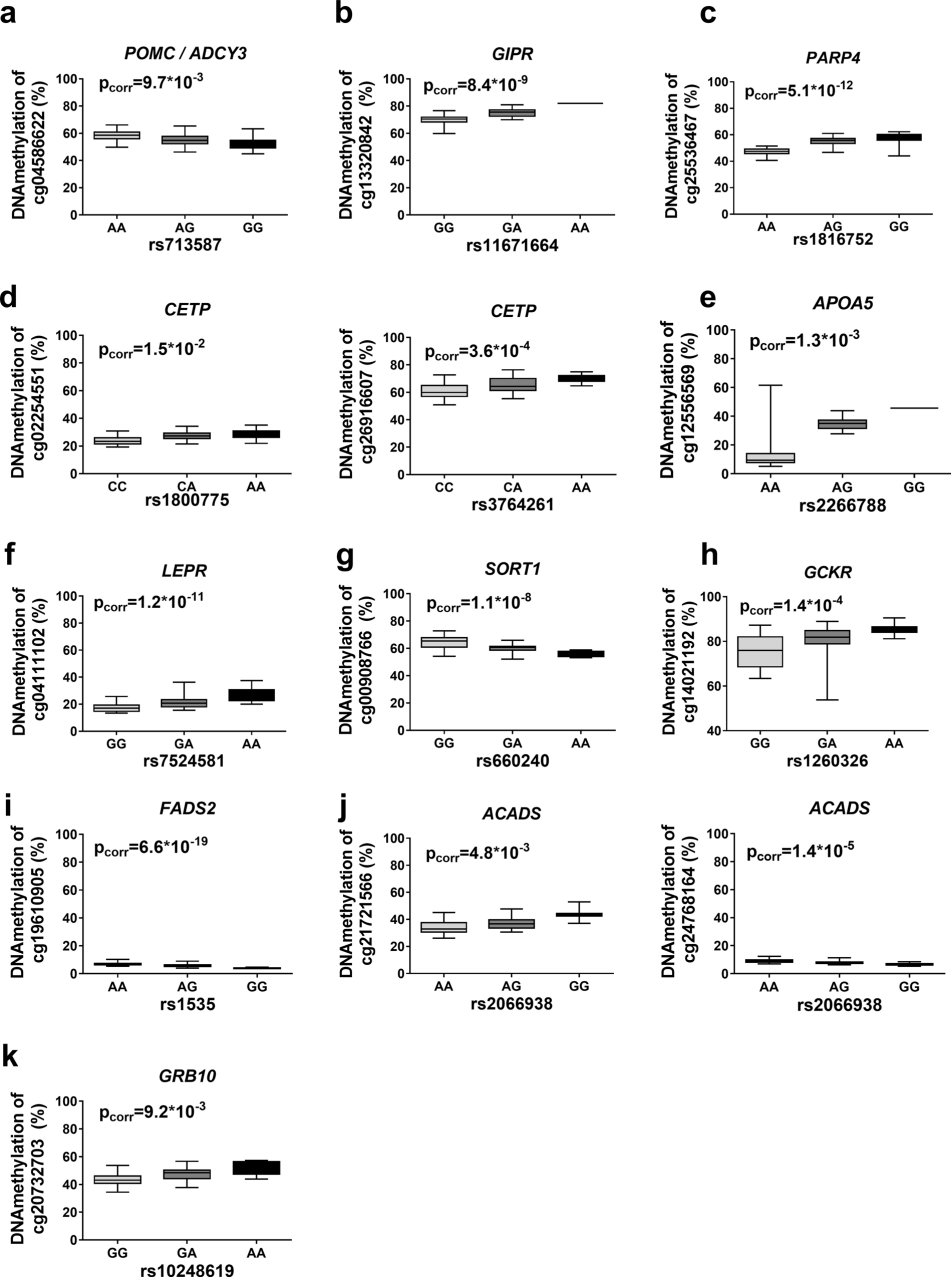
mQTLs in adipose tissue capture reported disease loci. Depiction of some identified mQTLs in adipose tissue of previously reported GWAS loci associated with obesity: (**a**) *POMC / ADCY3*, (**b**) *GIRP*, and (**c**) *PARP4*; lipid profiles, waist and metabolic syndrome: (**d**) *CETP*, (**e**) *APOA5*, (**f**) *LEPR*, (**g**) *SORT1*, (**h**) *GCKR* and (**i**) *FADS2*; and metabolic traits: (**j**) *ACADS* and (**k**) *GRB10*. *ADCY3* locus and *LEPR* loci were identified through proxy SNPs based on LD.

Numerous SNPs associated with obesity, type 2 diabetes and related traits have previously been identified by GWAS [1]. However, the molecular mechanisms explaining how most of these SNPs affect gene function and disease pathology remain scarce. We therefore tested if identified SNPs in significant mQTLs in adipose tissue overlap with loci previously reported to associate with obesity, type 2 diabetes or obesity/diabetes related traits in the GWAS catalog (p<10^−5^) [27]. Out of the SNPs significantly associated with DNA methylation in the *cis*-mQTL analysis and when taking proxy SNPs into account (R^2^>0.8, **see** Methods), 19,706 overall, we found 231 SNPs of significant mQTLs that overlapped with at least one of the 2138 reported disease or trait locus identified in the GWAS catalog (**S4 Table**), which constitutes 1.17% of *cis-*mQTL SNPs and 10.8% of GWAS catalog SNPs. Representative mQTLs for some of these loci are shown in **Fig 4A–4J**. These mQTLs include *POMC* and *LEPR*, which encode proopiomelanocortin and the leptin receptor, respectively. Mutations in both these genes have been associated with early onset obesity [28]. We also present mQTLs covering *GIPR* (encoding gastric inhibitory polypeptide receptor), *PARP4* (encoding poly(ADP-ribosyl)transferase-like 1 protein), *CEPT* (encoding cholesteryl ester transfer protein), *APOA5* (encoding apolipoprotein A5), *SORT1* (encoding sortilin 1), *GCKR* (encoding glucokinase regulator), *FADS2* (encoding fatty acid desaturase 2), *ACADS* (encoding acyl-CoA dehydrogenase) and *GRB10* (encoding growth factor receptor bound protein 10). SNPs in these loci have previously been associated with BMI, T2D and/or obesity- and lipid-related traits [29–36].

Of SNPs in significant *trans*-mQTLs, we found 4 SNPs overlapping with reported obesity loci in the GWAS catalog (**Fig 4K and S5 Table**).

### The impact of identified mQTLs on mRNA expression in human adipose tissue

**Fig 5:**
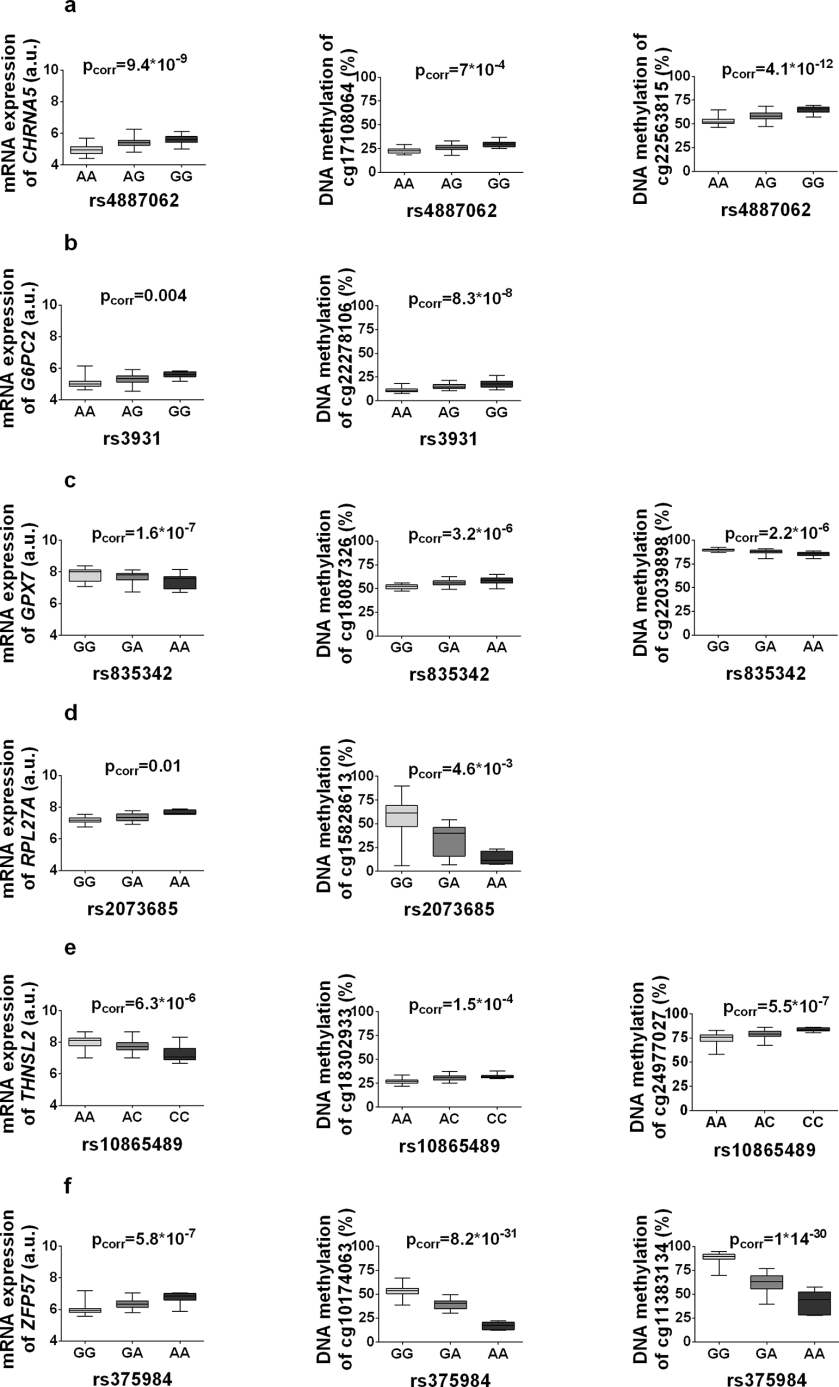
mQTLs affect gene expression in human adipose tissue. Significant mQTL SNP-CpG pairs where the SNP also shows significant association with gene expression in adipose tissue. The boxplots represent some identified mQTL SNPs and associations of the same loci with mRNA expression: (**a**) *CHRNA5*, (**b**) *G6PC2*, **(c)** *GPX7*, **(d)** *RPL27A*, **(e)** *THNSL2* and **(f)** *ZFP57*. Annotations for these mQTLs are included in S1 Table.

It is well established that mRNA expression is regulated by both genetic variation and DNA methylation independently [4,37]. However, the insights of how genetic and epigenetic variation interacts to influence gene expression remain limited. In order to study the impact of identified mQTLs on mRNA expression in human adipose tissue, we performed a follow-up eQTL analysis in 118 samples with available microarray expression data (out of original 119 samples). First, we related the 51,143 unique SNPs, showing significant association with DNA methylation in the *cis*-mQTL analysis, with mRNA expression of genes within 500kb (*cis*-distance). In the eQTL analysis of significant *cis*-mQTL SNPs, we identified 926 SNP-mRNA transcript pairs showing significant associations between genotypes and mRNA expression levels after correction for multiple testing (**see** Methods). These correspond to 635 unique SNPs and 86 unique genes, including *CHRNA5*, *G6PC2*, *GPX7*, *RPL27A*, *THNSL2* and *ZFP57* (**Table 3, Fig 5 and S6 Table**). *CHRNA5* encodes a nicotinic acetylcholine receptor subunit and SNPs in this locus have been associated with body weight in relation to tobacco use [38]. *G6PC2* encodes glucose-6-phosphatase catalytic subunit 2 and SNPs in this locus have been associated with glycemic traits [39]. *GPX7* encodes glutathione peroxidase 7 a protein involved in glutathione metabolism. *RPL27A* encodes Ribosomal protein L27A, which has been linked to human obesity [40]. *THNSL2* encodes threonine synthase like 2 and SNPs in this locus have been associated with obesity [41]. Additionally, *ZFP57* encodes a zink finger protein and DNA methylation and mutations in this locus are associated with transient neonatal diabetes [42].

**Table 3:** Number of significant eQTL results in human adipose tissue.

	eQTLs of *cis-*mQTL-SNPs	eQTLs of *trans-*mQTL-SNPs
SNP-mRNA transcript pairs	926	270	
Unique SNPs	635	89	
Unique mRNA transcripts	101	14	
Unique genes	86	10	

Only SNPs of significant mQTLs are included in the eQTL analysis.

SNPs of significant *cis*-mQTLs are regressed against mRNA expression of mRNA transcripts located in *cis* (≤ 500kb).

SNPs of significant *trans*-mQTLs are regressed against mRNA expression of all mRNA transcripts.

Significance threshold < 0.05 after correction for multiple testing.

Correction value for eQTL analysis for *cis-*mQTL-SNPs = 934,021

Correction value for eQTL analysis for *trans-*mQTL-SNPs = 33,326,082.

The 2,735 unique SNPs identified in the *trans*-mQTL analysis were also followed-up and related to mRNA expression of all analyzed genes. In the eQTL analysis of significant *trans*-mQTL SNPs, we identified 270 significant associations between genotypes and mRNA expression levels after correction for multiple testing (**see** Method), consisting of 89 unique SNPs and 10 unique genes *e*.*g*. *GSTT1*, *HLA-DQB1* and *ZFP57* (**Table 3 and S7 Table**).

### The impact of identified mQTLs on metabolic phenotypes

**Fig 6:**
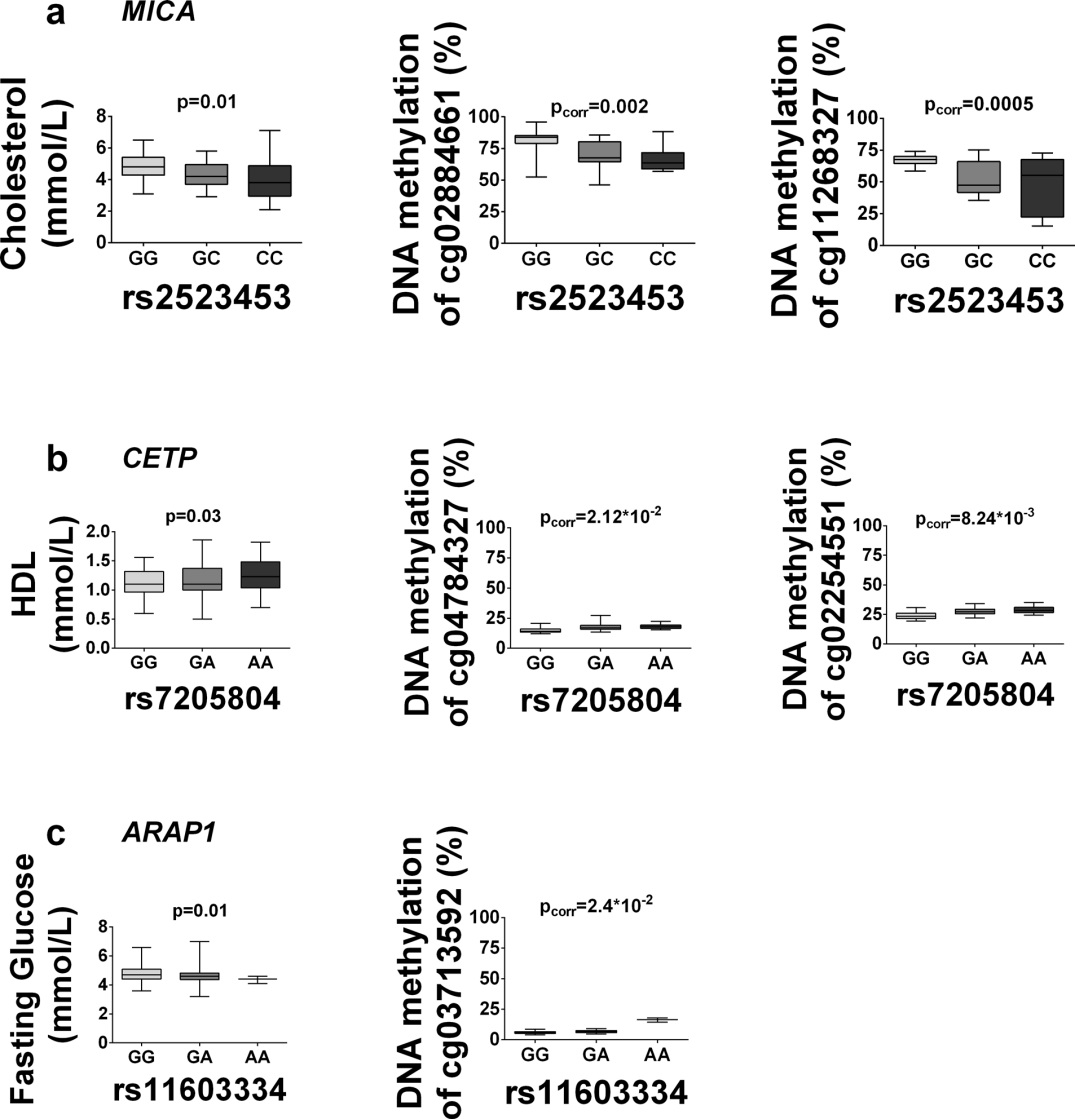
mQTLs in human adipose tissue affect metabolic phenotypes. The boxplots show significant mQTL SNPs associated with metabolic phenotypes in our study cohort with p<0.05, and associations of these loci with DNA methylation in adipose tissue for (**a**) *rs2523453*, (**b**) *rs7205804*, **(c)** *rs11603334*.

Given that adipose tissue contributes to whole body energy homeostasis by for instance insulin-stimulated glucose uptake, triglyceride storage and adipokine secretion, we investigated if the identified SNPs in significant mQTLs affect metabolic phenotypes in our study cohort. Identified mQTL SNPs were related to obesity measurements, glycemic traits and lipid levels in our study cohort of 119 Scandinavian men as well as looked-up in public available consortia data from the GIANT [43,44], MAGIC [36,45,46] and GLGC [47] consortia. Out of the significant *cis*-mQTLs, we found 62 SNPs associated with BMI, 185 with waist-hip ratio (WHR), 77 with fasting glucose, 62 with fasting insulin, 91 with homeostasis model of beta-cell function (HOMA-B), 49 with HOMA-IR, 146 with HbA1c, 85 with total cholesterol, 84 with triglycerides, 197 with HDL, 67 with LDL in both our study cohort and consortia data with the same direction of allele effects and with P≤0.05 (**S8 Table**). Several of these SNPs show genome-wide significance in GIANT, MAGIC or GLGC. Representative associations between genotype and some metabolic traits as well as DNA methylation in the 119 Scandinavian men are shown in **Fig 6A–6C.** The SNPs presented in **Fig 6**do also show genome-wide significance with respective trait in GLGC (*rs2523453*, cholesterol, p = 6.5*10^−08^ and *rs7205804*, HDL, p = 5.27^−675^) and MAGIC (*rs11603334*, fasting glucose, p = 2.9*10^−08^), respectively (**S8 Table)**.

**Fig 7:**
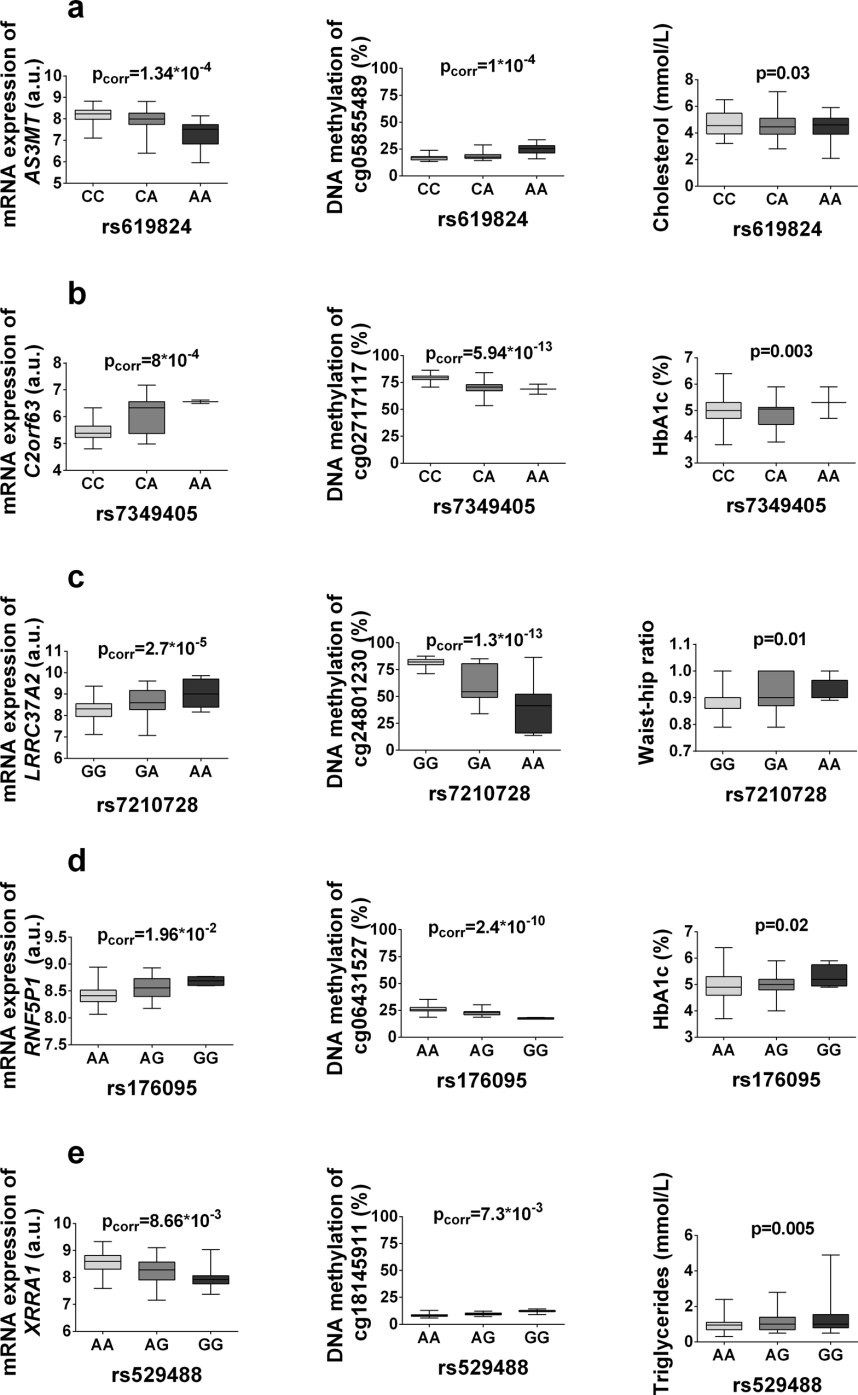
mQTLs/eQTLs in human adipose tissue affect metabolic phenotypes. Significant mQTL SNPs associated with both gene expression and a metabolic phenotype, with boxplots showing associations of some of these loci with DNA methylation, gene expression and metabolic traits: (**a**) *rs619824*, (**b**) *rs7349405*, (**c**) *rs7210728*, (**d**) *rs176095*, (**e**) *rs529488*.

Additionally, 8 loci detected in the overlap of the *cis*-mQTL and eQTL analysis were among those associated with metabolic phenotypes (**S8**and **S9 Tables**)**.** These data show the effect of interactions between common genetic variation and DNA methylation on gene expression and metabolic outcome (depiction presented in **Fig 7A–7E**, all overlapping SNPs presented in **S9 Table)**.

Of identified *trans*-mQTLs, we found 2 SNPs associated with BMI, 13 with WHR ratio, 6 with fasting glucose, 1 with fasting insulin, 2 with HOMA-IR, 42 with HbA1c, 6 with total cholesterol, 7 with triglycerides, 68 with HDL, and 4 with LDL in both our study cohort and consortia data with the same direction of allele effects and with P ≤0.05 (**S10 Table**).

Additionally, 2 of the identified *cis-*mQTL SNPS were previously found to be associated with C-reactive protein (CRP) levels (S11 Table).

Additionally, some of the identified *cis*- and *trans*- mQTLs are annotated to candidate genes for adipose-related traits. Out of the 157 loci previously implicated in lipid biology in GLGC consortium [47], 48 (30%) were found among 5,589 unique genes annotated to significant cis-mQTLs (S1 Table), and 4 among 375 unique genes annotated to significant trans-mQTLs (S2 Table).

### Causality inference test (CIT)–DNA methylation potentially mediates the genetic impact on metabolic phenotypes

**Fig 8:**
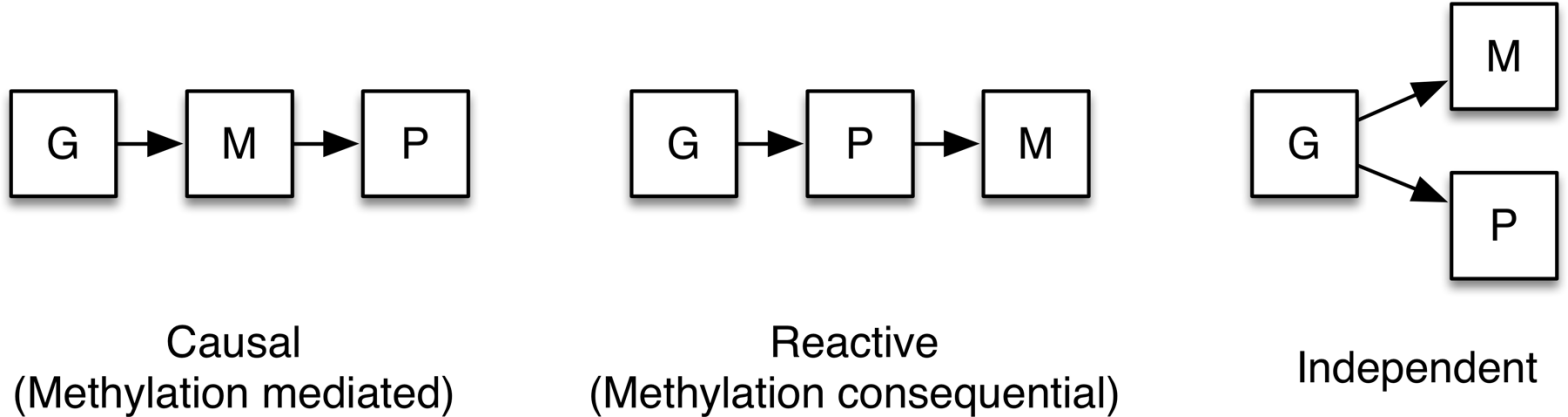
CIT in human adipose tissue. Possible relationship models between genotype as a causal factor (G), DNA methylation as the mediator factor (M) and metabolic phenotype as the phenotypic outcome (P).

We proceeded to evaluate the potential causality relationships between genotypes (G), DNA methylation (M) and phenotypic traits (P) using the CIT [21]. The possible relationships between these three factors are shown in **Fig 8.** The CIT was performed in our cohort of 119 Scandinavian men for identified SNP-CpG pairs in the mQTL analysis where the SNP also showed significant association with a metabolic phenotype in both our study cohort and publicly available consortia data with P ≤0.05. For *cis*-mQTLs, we identified 39 SNP-CpG pairs, corresponding to 35 unique SNPs and 22 unique CpGs, where SNP plays a causal role on metabolic phenotype, mediated by DNA methylation (**Table 4).** Out of these 39 SNP-CpG pairs, 1 pair was significantly associated with BMI, 2 for fasting glucose, 1 for fasting insulin, 1 for HOMA-B, 7 for HOMA-IR, 7 for HbA1c, 9 for cholesterol, 1 for triglycerides 5 for HDL and 5 for LDL (**Table 4**). Among the genes annotated to these SNP-CpG pairs, *CDK2AP1*, *HLA-DMA*, *MCM6*, *TCF19*, *CAMK1D* and *NEIL2* were found. None of the *cis*-mQTLs showed a reactive relationship between a SNP and a metabolic phenotype.

**Table 4:** Identified *cis*-mQTLs where DNA methylation potentially mediates the interactions between a genotype and a phenotype in human adipose tissue.

Chr	CpG Id	CpG Gene	CpG Gene Region	SNP Id	SNP Gene	G vs M p_corr_-value	Phenotype	G vs P p-value	CIT causal p-value
6	cg12929486	*SLC22A16*	TSS200	*rs2428190*	*SLC22A16*	1.30E-07	BMI	0.049	0.02
5	cg14825688	*LEAP2*	TSS1500	*rs39830*	*UQCRQ*	4.02E-06	Fasting glucose	0.015	0.03
5	cg14825688	*LEAP2*	TSS1500	*rs803217*	-	5.20E-05	Fasting glucose	0.018	0.03
2	cg01726273	*-*	Intergenic	*rs4853438*	*SNRPG*	3.60E-02	Fasting insulin	0.050	0.03
8	cg11123440		Intergenic	*rs12458*	*GATA4*	8.99E-03	HOMA-B	0.042	0.03
12	cg10240950	*C12orf76*	Body	*rs1027949*	*GIT2*	3.91E-02	HOMA-IR	0.036	0.05
12	cg10240950	*C12orf76*	Body	*rs10774978*	*TCHP*	3.91E-02	HOMA-IR	0.030	0.05
12	cg10240950	*C12orf76*	Body	*rs11068984*	*GIT2*	3.91E-02	HOMA-IR	0.017	0.05
10	cg26169081	*CAMK1D;CAMK1D*	Body;Body	*rs11257926*	*CAMK1D*	1.33E-03	HOMA-IR	0.021	0.04
10	cg26169081	*CAMK1D; CAMK1D*	Body;Body	*rs17152029*	*CAMK1D*	3.92E-04	HOMA-IR	0.003	0.01
10	cg26169081	*CAMK1D; CAMK1D*	Body;Body	*rs17152037*	*CAMK1D*	1.33E-03	HOMA-IR	0.005	0.04
12	cg10240950	*C12orf76*	Body	*rs2302689*	*ANKRD13A*	3.91E-02	HOMA-IR	0.030	0.05
7	cg17372657		Intergenic	*rs1880296*	*-*	2.69E-07	HbA1c	0.032	0.03
7	cg17372657		Intergenic	*rs2949170*	*-*	1.79E-04	HbA1c	0.034	0.03
7	cg17372657		Intergenic	*rs2949192*	*-*	9.02E-05	HbA1c	0.014	0.03
6	cg13561028	*SFTA2*	Body	*rs3130782*	*LOC100129065*	3.68E-09	HbA1c	0.002	0.01
6	cg13561028	*SFTA2*	Body	*rs3131934*	*-*	8.49E-08	HbA1c	0.002	0.04
16	cg04544033	*-*	Intergenic	*rs556179*	*-*	2.37E-02	HbA1c	0.014	0.03
6	cg13561028	*SFTA2*	Body	*rs7750641*	*TCF19*	1.27E-17	HbA1c	0.028	0.02
12	cg21745287	*ARL6IP4;OGFOD2*	TSS1500;3’UTR	*rs10846489*	*CDK2AP1*	4.91E-03	Cholesterol	0.013	0.04
12	cg07644039	*ARL6IP4;OGFOD2*	TSS1500;3’UTR	*rs10846489*	*CDK2AP1*	2.34E-02	Cholesterol	0.013	0.04
12	cg21745287	*ARL6IP4;OGFOD2*	TSS1500;3’UTR	*rs1109559*	-	4.84E-03	Cholesterol	0.011	0.03
12	cg07644039	*ARL6IP4;OGFOD2*	TSS1500;3’UTR	*rs1109559*	-	1.44E-02	Cholesterol	0.011	0.03
12	cg07644039	*ARL6IP4;OGFOD2*	TSS1500	*rs4275659*	*ABCB9*	2.24E-03	Cholesterol	0.010	0.02
12	cg21745287	*ARL6IP4;OGFOD2*	TSS1500;3’UTR	*rs6488868*	*SBNO1*	9.39E-04	Cholesterol	0.008	0.02
15	cg12371991	*-*	Intergenic	*rs6494591*	-	8.73E-03	Cholesterol	0.038	0.01
2	cg04490207	*-*	Intergenic	*rs6712567*	-	1.15E-02	Cholesterol	0.034	0.03
9	cg14341289	*FSD1L*	TSS1500	*rs885954*	-	1.81E-02	Cholesterol	0.041	0.00
8	cg00820056	*-*	Intergenic	*rs11787024*	*LY6H*	6.37E-03	Triglycerides	0.033	0.04
6	cg14833385	*HLA-DMA*	TSS1500	*rs1480380*	*-*	3.50E-17	HDL	0.007	0.03
2	cg01726273	*-*	Intergenic	*rs2921711*	*TIA1*	3.60E-02	HDL	0.037	0.04
2	cg07169764	*MCM6;MCM6*	1stExon;5'UTR	*rs309172*	*DARS*	4.06E-07	HDL	0.011	0.01
8	cg05875700	*ERICH1*	Body	*rs3735917*	*ERICH1*	3.17E-11	HDL	0.008	0.05
2	cg07169764	*MCM6;MCM6*	1stExon;5'UTR	*rs6750549*	*DARS*	4.06E-07	HDL	0.010	0.01
4	cg08029340	*MYL5*	Body	*rs11726338*	*PIGG*	9.63E-03	LDL	0.028	0.04
6	cg02525939	*-*	Intergenic	*rs4710698*	-	6.17E-07	LDL	0.007	0.04
6	cg04399728	*-*	Intergenic	*rs4710698*	-	2.69E-13	LDL	0.007	0.04
9	cg14341289	*FSD1L*	TSS1500	*rs885954*	-	1.81E-02	LDL	0.005	0.00
2	cg09644356	*-*	Intergenic	*rs940670*	-	2.94E-06	LDL	0.001	0.03

### Biological replication of mQTLs in human adipose tissue

To validate whether the results of mQTL analysis hold in an independent cohort, we also looked for overlap with a recent study also showing associations between genetic variation and DNA methylation in human adipose tissue [16]. While both studies analyzed DNA methylation using the Infinium HumanMethylation450 BeadChip, Grundberg *et al*. restricted their mQTL analysis to SNPs located within 100 kb from analyzed CpG sites [16] and therefore it was only possible to compare some of our results. It should also be taken into account that while our study included men, the study by Grundberg *et al*. included women and the two studies used different bioinformatic and statistical approaches, which may affect the possibility to replicate the results. Nevertheless, among our significant *cis*-mQTLs, we found that 5,468 CpG sites also stand under genetic control of SNPs in the study by Grundberg *et al*., and out of these 2,118 (38.6%) were associated with the same SNP in both their and our study [16].

Additionally, we recently performed an mQTL analysis in human pancreatic islets [20]. Here, we looked for overlap between the significant mQTLs identified in human adipose tissue of the 119 men and the mQTLs previously found in human pancreatic islets [20]. Among our significant *cis*-mQTLs in adipose tissue, 39,386 were also found in pancreatic islets **(S12 Table)**. Moreover, 1,852 significant *trans*-mQTLs overlapped between the two different tissues **(S13 Table)**.

### mQTLs in human adipose tissue do also show differential DNA methylation in patients with type 2 diabetes

We have previously identified CpG sites that are differentially methylated in adipose tissue from subjects with type 2 diabetes compared with non-diabetic controls [15]. However, it remains unknown if methylation of these sites may also be under genetic control. Therefore, we further tested if these CpG sites [15] overlap with our significant *cis* and *trans* mQTLs in human adipose tissue (**S1 and S2 Tables**). Interestingly, we discovered that 237 CpG sites among our significant *cis*-mQTLs and 7 CpG sites among our significant *trans*-mQTLs are also differentially methylated in adipose tissue from subjects with type 2 diabetes (**S14 Table**), suggesting that DNA methylation may mediate the genetic impact of type 2 diabetes.

### mQTLs in human adipose tissue overlap with CpG sites associated with BMI and HbA1c

We have previously identified CpG sites for which the adipose tissue methylation level associates with BMI and HbA1c [48]. Here, we examined if these CpG sites overlap with our *cis* and *trans*-mQTLs in human adipose tissue (**S1 and S2 Tables**). We found that 33,058 CpG sites previously identified as associated with BMI overlapped with 577 *cis* and 19 *trans* significant mQTLs in current study (**S15 Table**). Moreover, out of 711 CpG sites associated with HbA1c, 25 and 1 CpG site overlapped with significant *cis* and *trans* mQTLs respectively (**S15 Table).**

### mQTL analyses in adipose tissue of two sub-cohorts

Since the subjects in the four sub-cohorts included in this study differ in age and BMI, we performed a sub-analysis only including cohorts #1 and #2 as these subjects are phenotypically similar. Here, we detected 66,329 mQTLs in *cis* showing significant associations between genotype and the degree of DNA methylation after correction for multiple testing, corresponding to 36,909 unique SNPs and 11,788 unique CpG sites (**S16 Table**). Out of those 66,329 mQTLs, 63,714 (96%) overlapped with the analysis of all 4 cohorts.

In the *trans*-mQTL analysis, we identified 3,243 SNP-CpG pairs showing significant associations between genotypes and the degree of DNA methylation in adipose tissue after correction for multiple testing, corresponding to 1,865 unique SNPs and 538 unique CpG sites (**S17 Table**). Out of those 3,243 mQTLs, 2,919 (90%) were previously identified in the analysis of all 4 cohorts.

### mQTL analyses in adipose tissue without adjusting for BMI

In order to validate whether BMI as a covariate has a significant effect on a number of discovered mQTLs, we performed a mQTL analysis of all 4 study cohorts without BMI as a covariate. Overall, we detected 102,467 significant *cis* mQTLs corresponding to 51,435 unique SNPs and 15,267 unique CpG sites. Out of those, 99,661 (97.2%) were also identified in the analysis where BMI was included as a covariate (**S18 Table**). In *trans*, we discovered 5,435 significant mQTLs, corresponding to 608 unique CpG sites and 2,765 unique SNPs, where 5,272 (97%) were also identified in the main mQTL analysis (**S19 Table**).

### Associations between DNA methylation and mRNA expression in human adipose tissue

We finally tested the direct association between DNA methylation and gene expression in human adipose tissue by performing a linear regression between individual mRNA transcripts and DNA methylation of CpG sites in cis (500 kb up- and 100 kb downstream of respective gene) including age, BMI and study cohort as covariates. We found significant associations between DNA methylation and mRNA expression for 546 combinations (FDR<5%), consisting of 473 unique CpG sites and 194 unique mRNA transcripts (**S20 Table**), which are annotated to 173 genes.

In addition, we found that 262 CpG sites among our significant cis-mQTLs and 13 among our significant trans-mQTLs overlapped with methylation sites associated with mRNA expression in adipose tissue (**S20 Table**).

## Discussion

The present study highlights the importance of genome-wide interactions between genetic and epigenetic variation and its role in human metabolism. Using CIT tests, we could for the first time identify adipose tissue methylation-mediated relationships between genotype and metabolic phenotypes, including lipid and glucose traits. Importantly, these data demonstrate how genetic variants may mediate their effects on metabolic traits via altered DNA methylation in human adipose tissue. Additionally, numerous identified mQTL-SNPs cover previously identified GWAS loci for obesity, lipid and diabetes related traits e.g. *POMC*, *GIPR*, *GRB10*, *FADS2*, *SORT1* and *APOA5*.

Multiple SNPs identified through GWAS associate with complex metabolic disease including obesity and type 2 diabetes [29,31,43,49–52]. However, the effect sizes of common variants influencing these diseases are often modest and in total they only explain small proportions of the estimated genetic predispositions to the diseases. Epigenetic factors such as DNA methylation have also been shown to be involved in the pathogenesis of various metabolic diseases [7,9,15,29,53–60]. However, studies examining the genetic regulation of inter-individual variation in DNA methylation and its contribution to metabolic outcomes are scarce but would likely give new insights to the field. Here, we performed a genome-wide mQTL analysis looking at both *cis* and *trans* effects of genetic variation on DNA methylation in human adipose tissue. To further link identified mQTLs with biological functions, we performed follow-up analyses of significant mQTL SNPs with gene expression in adipose tissue and metabolic phenotypes in our study cohort. We also looked for overlap with disease loci reported to associate with obesity and diabetes related traits in GWAS. All together, we found 101,911 SNP-CpG pairs in *cis* and 5,342 SNP-CpG pairs in *trans* showing significant associations between genotype and DNA methylation in adipose tissue demonstrating a strong genetic impact on DNA methylation in human adipose tissue. Our data are in line with previous mQTL analyses, which also show strong interactions between genetic and epigenetic variation [16–20,61], and in concordance, we found an enrichment of *cis*-mQTLs in a short distance window between associated SNPs and CpG sites. However, while most previous mQTLs have been limited to studying promoter regions [17–19] or *cis* interactions [16], we can for the first time present mQTL results in adipose tissue looking at both *cis* and *trans* effects in most genomic regions and genes. Interestingly, we observe a higher than expected number of methylation sites in significant mQTLs located in intergenic regions, in the gene body and outside of CpG islands. This observation is in line with previous studies showing that differentially methylated sites in response to environmental or genetic factors to a higher extent than expected are located outside CpG islands or within intergenic and gene body regions [10,16]. It may be that promoter regions are rich in CpG islands which are hypomethylated and are more evolutionary conserved based on their biological function, meanwhile non-CpG islands are more methylated and dynamic [6,62–64]. Interestingly, we demonstrate for the first time an enrichment of significant mQTLs in adipose tissue on chromosome 6. This chromosome possesses a highly polymorphic gene region coding the HLA complexes which are known to be implicated in several autoimmune disorders and inflammation processes [23,24]. Numerous loci identified in the *cis*- and *trans*-mQTL analysis, as well as genes in the eQTL follow-up analysis, are linked to the *HLA* genes. Based on this finding, we investigated the link between mQTLs on chromosome 6 and a measure of inflammation e.g. i.e. CRP levels. Interestingly, we found that 2 SNPs in significant mQTLs cover GWAS loci associated with CRP levels (**S11 Table**). However, none of them was located on chromosome 6 [65].

Genetic association studies have improved our understanding of the biological basis of metabolic disease [66]. Nevertheless, the effect of numerous reported obesity and diabetes SNPs on target genes or biology still remains unknown. Investigating the genetic control of variation in DNA methylation may improve our understanding of biological processes and linking loci to tissue dependent phenotypes and diseases. Elevating, we found that several SNPs associated with DNA methylation show impact on metabolic phenotypes in the studied cohort, including obesity measurements, glucose- and insulin traits, as well as lipid profiles. The effect of mQTLs on molecular phenotypes was further supported by independent replication in consortia data of obesity measurements from GIANT [43,44], glucose traits from MAGIC [36,45,46] and lipid profiles from GLGC [47]. Although mQTL SNPs were only showing nominal association to metabolic phenotypes in our study cohort, the overlap and replication in independent studies, based on consortium data, support effects of these SNPs on biological function. Indeed, several of these SNPs show genome-wide significance in previous GWAS [47,66–68]. These include SNPs associated with cholesterol levels and annotated to *ANKRD31* (ankyrin repeat domain 31), HDL levels and annotated to *CELSR2* (cadherin, EGF LAG seven-pass G-type receptor 2) as well as fasting plasma glucose levels and annotated to *ARAP1* (ankyrin repeat and PH domain 1).

Given that SNPs affect DNA methylation and that DNA methylation is a dynamic process that may change in response to environmental factors and affects phenotype transmission [10,69], it may be possible that the SNP effect on DNA methylation levels, and indirectly on metabolic phenotypes, may change under different environmental conditions. It is hence possible that some of the identified mQTL SNPs overlapping with consortium data may have escaped detection to disease phenotypes in previous GWAS studies since DNA methylation levels was not considered. This form of gene-environment interactions could potentially affect the SNPs impact on disease risk. Indeed, our previous data, where we identified a SNP that introduces a CpG site in the promoter of *NDUFB6*, support this hypothesis [60]. Here, we showed that while elderly carriers of the genotype that introduces a CpG site had a high degree of methylation in the SNP-CpG site together with decreased skeletal muscle *NDUFB6* expression and decreased glucose uptake, young carriers had low degree of methylation in the SNP-CpG site together with increased skeletal muscle *NDUFB6* expression and no effect on glucose uptake. Together, this study demonstrates a clear interaction between genetic, epigenetic and non-genetic factors. Additionally, genetic variation may carry inheritance of epigenetic variation and thereby have an impact on the heritability of human diseases and may explain some of the missing heritability of human complex diseases. Furthermore, we also found that several SNPs associated with DNA methylation in adipose tissue overlapped directly or via proxy SNPs to previously reported disease loci of obesity related traits, including *CETP* and *FADS2*, which are both known to be associated with total cholesterol, LDL, HDL and triglyceride levels [47] These data support that genetic and epigenetic variation together influence metabolic phenotypes and disease risk in humans.

In order to provide further insights into mechanisms of genetic and epigenetic interaction and its impact on regulation of metabolic phenotypes, we used the CIT [21,70]. We discovered 39 significant mQTLs where DNA methylation represents the mediator between genetic loci and a metabolic trait. One of these mQTLs SNPs is associated with HDL regulation through DNA methylation of a CpG site annotated to *MCM6*. This is an MCM (minichromosome maintenance) complex gene that previously has been shown to affect total cholesterol levels [71]. Among other genes identified in the CIT analysis were *TCF19*, which has been associated with type 1 diabetes through GWAS [72], and *CAMK1D*, which has been associated with type 2 diabetes [73] This supports the role of DNA methylation as a direct mediator between genetic variation and metabolic phenotypes. However, while the majority of significant *cis*-mQTL SNP-CpG pairs were found to have independent effects on the analyzed phenotypes, the independence cannot be concluded due to some limitations in our analysis. First, only a few phenotypes were considered in the course of the analysis, and it might require other phenotypes to discover all cause–effect relationships between SNPs, methylation and metabolic phenotype. Second, as CIT only considers one SNP and one CpG site at a time, more complex interactions involving several SNPs and or CpGs can be missed, which suggests that more sophisticated analytical methods should be developed. An additional drawback of our study is the small sample size relative to the number of statistical comparisons. As the number of analyzed SNP–CpG pairs is in the order of 10^11^, only the strongest interaction effects can be detected by means of our mQTL analysis. To reduce the number of type 2 errors during multiple testing procedures, we implemented a modified Bonferroni correction method, which took into account a linkage disequilibrium dependency between analyzed SNPs. Additionally, we performed eQTL and CIT analysis only on the data that was shown to be significant in the mQTL analysis, thus again significantly reducing number of performed statistical tests. While eQTL analyses have been used to identify causal genetic variants for metabolic disease [74], here we provide the first CIT analysis of genetic variation, DNA methylation in adipose tissue and metabolic traits. Importantly, this analysis demonstrates how genetic variants mediate their effects on metabolic traits (e.g. BMI, cholesterol, HDL, HbA1c and HOMA-IR) via altered DNA methylation in human adipose tissue.

Interestingly, SNPs throughout the genome may introduce or delete CpG sites and thereby affect the possibility for DNA methylation to take place [22]. These so called CpG-SNPs are likely to show strong correlations with the degree of methylation in the SNP site. Indeed, here we found 447 CpG-SNPs associated with DNA methylation in adipose tissue.

Furthermore, we were able to replicate numerous of our unique CpG sites of significant *cis*-mQTLs in a study by Grundberg *et al*. [16] confirming the biological importance of our results. While both our study and Grundberg *et al*. performed mQTL analyses in human adipose tissue using the Illumina 450K array for DNA methylation and thereby comparable, divergence in *cis* boundary, sex and correction methods for multiple testing may explain some of the different results between the studies. It should also be noted that 39,386 of our significant *cis*-mQTLs in human adipose tissue were previously also identified in human pancreatic islets [20]. While this finding shows that some SNPs affect the DNA methylation pattern in multiple tissues, additional mQTL studies using the 450k array are needed in other tissues to test if the same associations are seen there.

We provide for the first time a combined genome-wide *cis*- and *trans*-mQTL analysis in human adipose tissue covering most genes and genomic regions. Our study demonstrates that interactions between genetic and epigenetic variation influences gene expression, molecular phenotypes and metabolic traits related to complex diseases in humans. We also provide details on potential causal relationships between genetic and epigenetic variation on metabolic phenotypes. Thus, DNA methylation variation may be of high importance in genetic association studies and may improve our understanding of molecular pathways in the context of complex human metabolic diseases.

## Materials and Methods

### Study samples and phenotypes

This study includes a total of 119 Scandinavian men without known disease. Their characteristics are presented in **Table 1**. The cohort includes subjects from four sub-cohorts, all previously described [15,48,75–77] and with DNA available from subcutaneous adipose tissue biopsies taken in the fasted state. The characteristics of the four sub-cohorts are presented separately in **S21 Table.** All study participants underwent a physical examination including measurements of BMI, waist and WHR. Moreover, blood sampling for analysis of lipids, glucose and insulin were done during the fasting state. Written informed consent was obtained from all participants and the research protocols were approved by the local human research ethics committees: Dnr 13/2006 (Lund University), Dnr 461/2006 (Lund University), KA 03129gm (Köpenhavns AMT). While three of sub-cohorts are intervention studies [75–77], one sub-cohort is a case-control cohort [15]. Only baseline samples from healthy subjects were included in this study.

### Genotype data

Genotyping was performed in DNA extracted from blood of the 121 Scandinavian men using Illumina HumanOmniExpress BeadChip, which is a genome-wide array covering 731,412 SNPs, together with the iScan system (Illumina, San Diego, CA, USA). Genomic DNA was extracted from blood using the Gentra Puregene Blood Kit (Qiagen, Hilden, Germany). Genotypes were called using GenomeStudio® software (Illumina). All subjects passed call rate threshold of > 98%. Sex discrepancy between reported sex and genotypic sex based on X-chromosome heterozygosity was detected for two subjects and these subjects were excluded from subsequent analyses. No subjects were found to be population outliers based on a population stratification test. SNPs were excluded if missing calls > 5%, Hardy-Weinberg Equilibrium p-value < 0.001 and minor allele frequency < 0.05. Overall, 592,794 SNPs for 119 subjects passed quality control and were used for subsequent analyses. All genotype data were analyzed using Plink software (http://pngu.mgh.harvard.edu/purcell/plink/) [78].

### DNA methylation data

Genome-wide DNA methylation profiling was performed in genomic DNA extracted using Qiagen DNA extraction kits (Qiagen) from adipose tissue from 119 Scandinavian men using the Infinium HumanMethylation450 BeadChip (Illumina). The DNA methylation array targets 485,577 probes across the genome, covering 99% of RefSeq genes and 96% of CpG islands. Genomic DNA (500 ng) from adipose tissue was bisulfite treated using the EZ DNA methylation kit (Zymo Research, Orange, CA, USA). DNA methylation analysis of bisulfite treated DNA was carried out with Infinium® assay following the standard Infinium HD Assay Methylation Protocol Guide (Part #15019519). BeadChips were scanned with Illumina iScan and raw data was imported to the GenomeStudio Methylation module software for calculation of methylation scores represented as methylation β-values. In sample quality control, all samples passed GenomeStudio quality control steps for bisulfite conversion efficiency, staining, hybridization, extension and specificity.

Individual probes with a mean Illumina detection p-value > 0.01 were considered not detected and subsequently excluded from further analysis. Non-CpG methylation probes and SNP-probes included on the array were also filtered out. After these quality control steps and after filtering DNA methylation data, 477,891 CpG sites remained for all included samples. Before further analysis, the DNA methylation data was exported from GenomeStudio and subsequently analyzed using Lumi package from Bioconductor [79]. Extracted methylation data were then converted from β-values to M-values [80], $M={log}_{2}\left(\frac{\max\left(M,0\right)+1}{\max\left(U,0\right)+1}\right)$, where M and U are methylated and unmethylated channel intensities, respectively. The data was further background corrected and quantile normalized using lumi package [81]. To correct for batch effects, COMBAT normalization method [82] was used.

### mRNA expression data

Genome-wide mRNA expression profiling using the whole-transcript GeneChip® Human Gene 1.0 ST Array (Affymetrix, Santa Clara, CA, USA) following the Affymetrix standard protocol was performed in RNA extracted from the subcutaneous adipose tissue biopsies of 118 out of 119 Scandinavian men using miRNeasy kit followed by the RNeasy MiniElute Cleanup Kit (Qiagen) or using the RNeasy Lipid Tissue Mini Kit (Qiagen). The array data was background corrected, quantile normalized and summarized with robust multichip average (RMA) procedure using oligo package [83] from Bioconductor. Normalized dataset was batch corrected using COMBAT [82]. In total, mRNA expression of 28,779 transcripts was obtained for subsequent analyses.

### mQTL analysis

Associations between SNPs and DNA methylation of CpG sites were modeled as a linear relationship using DNA methylation levels as a dependent variable, SNP genotypes encoded as 0, 1 or 2 according to number of minor alleles. Due to the fact that both BMI and age can affect DNA methylation and, therefore, the association between SNP and DNA methylation, age, BMI and the sub-cohort were included as covariates. Calculations of associations were performed using the MatrixEQTL library for R programming language [84].

To distinguish between local (*cis*) and distant (*trans*) mQTLs a distance less or equal to 500 kb between a SNP and CpG site was used to define *cis*-mQTLs. All remaining SNP-CpG pairs were considered *trans*-mQTLs. In total we found 283,290,917,454 CpG–SNP pairs in the dataset, where 112,842,462 pairs were defined to be located in *cis* and 283,178,074,992 in *trans*. The *cis*- and *trans*-mQTL analyses were performed separately. In order to correct for multiple testing, p-value significance threshold was set, accounting for number of tests performed as well as the dependency of linkage disequilibrium (LD) between SNPs. LD-based SNP pruning was used to take into account the linkage dependency of SNPs that are run against the same quantitative trait locus in the mQTL analysis by calculating the number of independent tests based on r^2^<0.9 for the SNPs. In the *cis*-analysis, LD based pruning of SNPs within a distance of 500 kb from a CpG site was performed by pairwise-tagging (r^2^<0.9) and the total sum of all tag SNPs connected to each CpG site was used as correction value when correcting for multiple testing. LD calculations were performed using R trio package [85]. The correction value for the *trans*-analysis was calculated as the total number of analyzed CpG sites multiplied by the number of tag SNPs in the whole dataset (pairwise-tagging r^2^<0.9) and subtracted by the correction value for the *cis*-analysis. Significance threshold was set to p<0.05 after correction for multiple testing. All SNPs connected to each CpG site after LD-based pruning were summed and the remaining number of 104,023,091 SNP-CpG pairs was used as correction value for multiple testing in *cis*. This resulted in a significance threshold of 0.05/104,023,091 = 4.8x10^-10^ in *cis*. In the *trans*-mQTL analysis, after LD-based pruning, 211,781,637,483 SNP-CpG pairs remain and this number was used as correction value for multiple testing. This resulted in a significance threshold of 0.05/211,781,637,483 = 2.3x10^-13^ in *trans*.

### Impact of significant mQTL SNPs on mRNA expression

The relationship between SNPs found to be significantly associated with DNA methylation in the mQTL analysis and mRNA expression was tested in 118 of the men included in the study using a linear regression model with mRNA expression as a dependent variable, SNP genotypes encoded as 0, 1 or 2 according to number of minor alleles, and age, BMI and sub-cohort as covariates. Significant SNPs identified in the *cis*-mQTL analysis were only related to mRNA expression transcripts of genes located within 500 kb from respective SNP (*cis*). Significant SNPs identified in the *trans*-mQTL were related to mRNA expression transcripts of all analyzed genes. In total, 1,164,807 SNP-mRNA transcript combinations were found for significant *cis*-mQTLs, and 78,710,565 SNP-mRNA transcript combinations were found for significant *trans*-mQTLs. Correction value for multiple testing in the eQTL analysis was then calculated in similar way as for the mQTL analysis taking LD-based SNP pruning (r^2^<0.9) into account. In the eQTL analysis of significant *cis*-mQTL SNPs, the number of LD pruned SNPs (r^2^<0.9) to each mRNA transcript within 500 kb were summed up and used as the correction value for multiple testing. After LD-based pruning, 934,021 SNP-mRNA transcripts remain. This resulted in a significance threshold of 0.05/934,021 = 5.4x10^-8^ in *cis*. In the eQTL analysis of significant *trans*-mQTL SNPs, the correction value for multiple testing was calculated as the number of all *trans*-mQTL SNPs pruned for LD (r^2^<0.9) multiplied by total number of analyzed mRNA transcripts giving a remaining number of 33,326,082 SNP-mRNA transcripts. This resulted in a significance threshold of 0.05/33,326,082 = 1.5x10^-9^ in *trans*.

### Impact of mQTL SNPs on metabolic phenotypes

The impact of identified SNPs in significant mQTLs on the following phenotypes; BMI, WHR, cholesterol, triglycerides, HDL, LDL, fasting glucose, fasting insulin, HOMA-B, HOMA-IR and HbA1c, was tested in 119 Scandinavian men included in this study. Associations between identified SNPs in the significant mQTLs and metabolic phenotypes were modeled as a linear relationship using metabolic phenotypes as the dependent variable, SNP genotypes encoded as 0, 1 or 2 according to number of minor alleles, and age and sub-cohort included as covariates in all the analyses. BMI was also included as a covariate when analyzing associations between SNPs and fasting glucose, fasting insulin, HOMA IR, HOMA-B and HbA1c. Traits for fasting insulin, HOMA-B and HOMA-IR have been naturally log transformed in the study cohort before analyses. Identified mQTL SNPs showing association to a metabolic phenotype in our study cohort (p<0.05), were also looked-up in public available GWAS data from the GIANT consortium [43,44], MAGIC investigators [36,45,46] and GLGC consortium [47], for respective trait. SNPs showing association to a metabolic phenotype with the same allelic effect sign and with p-value <0.05 in both our study cohort and consortia data were considered detected.

### Overlap between mQTL SNPs and public available GWAS data

The catalog of published GWAS data was used to search for SNPs reported to be associated with obesity, type 2 diabetes and related metabolic traits (p<10^−5^). To increase reference coverage for overlap between datasets of identified mQTL SNPs and identified SNPs reported in GWAS catalog, a SNP annotation and proxy (SNAP) search [86] was performed to identify SNPs in LD with the identified mQTL SNPs. The proxy search was based on pairwise LD calculations of genotype data from the 1000 Genomes project of the CEU population panel with r^2^>0.8 and distance limit of 500 kb from the query SNP.

### Causal Inference Test (CIT)

The CIT was used to test if DNA methylation is a mediator between genotype variation and a phenotypic trait [21]. The causality can be inferred if all of the following are true: 1) G and M are associated, 2) G and P are associated, 3) G is associated with M|P and 4) G is independent of P|M, where G is a genotype marker, M is a DNA methylation measure and P is a phenotypic trait, provided that G is randomized [21]. Causal role of DNA methylation is inferred if p-value for causal relationship hypothesis is less than 0.05.

### Statistical analysis

Data were analyzed using linear regression models, Pearson chi-squared test or Fisher's exact test. All statistical calculations were performed using R programming language [87]. Results are expressed as Box and Whiskers plots. Pathway analysis using WebGestalt [26].

## Supporting Information

S1 Table Identified *cis*-mQTLs. Sheet a: Identified *cis*-mQTL SNP-CpG pairs, including chromosomal location and relation to CpG islands and gene regions. Sheet b: SNP-CpG pairs where SNP is located in either C or G of the CpG site, so called CpG-SNPs. Sheet c: Additional annotation data for SNPs present in sheet a, based on HumanOmniExpress-12v1_J_Gene_Annotation_build37 (Illumina). Sheet d: Additional annotation data for CpGs present in sheet a, based on Infinium HumanMethylation 450 BeadChip [25] and probe cross-reactivity info as reported by Chen et al [88].
(XLSX)

S2 Table Identified *trans*-mQTLs. Sheet a: Identified *trans*-mQTL SNP-CpG pairs, including statistical results of associations and chromosomal location and relation to CpG islands and gene regions. Sheet b: Additional annotation data for SNPs present in sheet a, based on HumanOmniExpress-12v1_J_Gene_Annotation_build37 (Illumina). Sheet c: Additional annotation data for CpGs present in sheet a, based on Infinium HumanMethylation 450 BeadChip [25] and probe cross-reactivity info as reported by Chen et al [88]
(XLSX)

S3 Table KEGG pathways identified among genes annotated to significant *cis* mQTL CpG sites. Sheet a: **KEGG** pathways enriched among genes annotated to CpG sites from significant *cis-*mQTLs. Sheet b: **KEGG** pathways enriched among genes annotated to CpG sites from significant *trans-*mQTLs.
(XLSX)

S4 Table Identified *cis*-mQTL SNPs that are also reported as disease SNPs in GWAS catalog [27]. SNPs that are found directly in the catalog are marked with grey, and ones that are found to be in LD with GWAS catalog SNPs with white. LD proxy analysis performed using SNAP (1000 Genomes project, CEU population panel, r2 > 0.8, distance limit 500kb) [86].
(XLSX)

S5 Table Identified *trans*-mQTL SNPs that are also reported as disease SNPs in GWAS catalog [27]. SNPs that are found directly in the catalog are marked with grey, and ones that are found to be in LD with GWAS catalog SNPs with white. LD proxy analysis performed using SNAP (1000 Genomes project, CEU population panel, r2 > 0.8, distance limit 500kb) [86].
(XLSX)

S6 Table Identified significant *cis-*mQTL SNPs that also show associations with gene expression. Sheet a: Identified *cis* SNP-mRNA transcript pairs, including statistical results of associations and gene assignment for mRNA transcripts. Only pairs with p-value < 0.05 after multiple testing corrections are included. Sheet b: Additional annotation data for SNPs present in sheet a, based on HumanOmniExpress-12v1_J_Gene_Annotation_build37 (Illumina). Sheet c: Additional annotation data for probesets present in sheet a. Annotations are based on NetAffx transcript cluster data for HuGene-1_0-st array (Affymetrix).
(XLSX)

S7 Table Identified significant *trans*-mQTL SNPs that also show associations with gene expression. Sheet a: Identified *trans* SNP-mRNA transcript pairs, including statistical results of associations and gene assignment for mRNA transcripts. Sheet b: Additional annotation data for SNPs present in sheet a. Sheet c: Additional annotation data for probesets present in sheet a. Annotations are based on NetAffx transcript cluster data for HuGene-1_0-st array (Affymetrix).
(XLSX)

S8 Table Identified *cis*-mQTL SNPs that show association with metabolic phenotypes in the study cohort (p<0.05) and are also identified in MAGIC, GIANT, or GLGC consortia (p<0.05). Excel table representing *cis*-mQTL SNPs that are identified in MAGIC, GIANT, or GLGC consortia (p<0.05) overlapping with SNPs that show association with metabolic phenotypes in the study cohort (p<0.05). Sheet a: SNPs associated with BMI in study cohort and GIANT consortium [44]. Sheet b: SNPs associated with Waist-hip ratio in study cohort and GIANT consortium [43]. Sheet c: SNPs associated with Fasting glucose in study cohort and MAGIC consortium [45]. Sheet d: SNPs associated with Fasting insulin in study cohort and MAGIC consortium [45]. Sheet e: SNPs associated with HOMA-B in study cohort and MAGIC consortium [45]. Sheet f: SNPs associated with HOMA-IR in study cohort and MAGIC consortium [45]. Sheet g: SNPs associated with HbA1c in study cohort and MAGIC consortium [46]. Sheet h: SNPs associated with Total cholesterol in study cohort and GLGC consortium [47]. Sheet i SNPs associated with Triglycerides in study cohort and GLGC consortium [47]. Sheet j: SNPs associated with HDL in study cohort and GLGC consortium [47]. Sheet k: SNPs associated with LDL in study cohort and consortium GLGC [47].
(XLSX)

S9 Table Significant *cis-*mQTL/eQTL SNPs that show association with metabolic phenotypes in the study cohort and are also identified in MAGIC, GIANT, or GLGC consortia (p<0.05). (XLSX)

S10 Table Identified *trans*-mQTL SNPs that show association with metabolic phenotypes in the study cohort (p<0.05) and are also identified in MAGIC, GIANT, or GLGCR consortia (p<0.05). Sheet a: SNPs associated with BMI in study cohort and GIANT consortium [44]. Sheet b: SNPs associated with Waist-hip ratio in study cohort and GIANT consortium [43]. Sheet c: SNPs associated with Fasting glucose in study cohort and MAGIC consortium [45]. Sheet d: SNPs associated with Fasting insulin in study cohort and MAGIC consortium [45]. Sheet e: SNPs associated with HOMA-B in study cohort and MAGIC consortium [45]. Sheet f: SNPs associated with HOMA-IR in study cohort and MAGIC consortium [45]. Sheet g: SNPs associated with HbA1c in study cohort and MAGIC consortium [46]. Sheet h: SNPs associated with Total cholesterol in study cohort and GLGC consortium [47]. Sheet i SNPs associated with Triglycerides in study cohort and GLGC consortium [47]. Sheet j: SNPs associated with HDL in study cohort and GLGC consortium [47]. Sheet k: SNPs associated with LDL in study cohort and consortium GLGC [47].
(XLSX)

S11 Table Significant *cis-*mQTL SNPs associated with CRP in Denghan et al. [65]. (XLSX)

S12 Table Significant *cis*-mQTL SNP-CpG pairs that are also reported to show significant associations in Olsson et al. [20]. (XLSX)

S13 Table Significant *trans*-mQTL SNP-CpG pairs that are also reported to show significant associations in Olsson et al. [20]. (XLSX)

S14 Table Significant *cis*- and *trans-* mQTL CpG sites that are also reported to show differential methylation in Nilsson et al. [15]. Sheet a: Identified *cis-*mQTL CpG sites that are reported in Nilsson et al. [15]. Sheet b: Identified *trans-*mQTL CpG sites that are reported in Nilsson et al. [15]. Sheet c: Additional annotation data for CpGs present in sheet a, based on Infinium HumanMethylation 450 BeadChip. [25]. Sheet d: Additional annotation data for SNPs present in sheet a, based on HumanOmniExpress-12v1_J_Gene_Annotation_build37 (Illumina).
(XLSX)

S15 Table Significant *cis*- and *trans-* mQTL CpG sites that are also reported to show significant associations with BMI and HbA1c in Rönn et al. [48]. (XLSX)

S16 Table *cis*-mQTLs identified in the analysis of subcohorts 1 and 2. Sheet a: Identified *cis*-mQTL SNP-CpG pairs, including chromosomal location and relation to CpG islands and gene regions. Sheet b: SNP-CpG pairs where SNP is located in either C or G of the CpG site, so called CpG-SNPs. Sheet c: Additional annotation data for SNPs present in sheet a, based on HumanOmniExpress-12v1_J_Gene_Annotation_build37 (Illumina). Sheet d: Additional annotation data for CpGs present in sheet a, based on Infinium HumanMethylation 450 BeadChip [25] and probe cross-reactivity info as reported by Chen et al [88].
(XLSX)

S17 Table *trans*-mQTLs *cis*-mQTLs identified in the analysis of subcohorts 1 and 2. Sheet a: Identified *trans*-mQTL SNP-CpG pairs, including statistical results of associations and chromosomal location and relation to CpG islands and gene regions. Sheet b: Additional annotation data for SNPs present in sheet a, based on HumanOmniExpress-12v1_J_Gene_Annotation_build37 (Illumina). Sheet c: Additional annotation data for CpGs present in sheet a, based on Infinium HumanMethylation 450 BeadChip [25] and probe cross-reactivity info as reported by Chen et al [88].
(XLSX)

S18 Table *cis*-mQTLs identified without BMI as a covariate. Sheet a: Identified *cis*-mQTL SNP-CpG pairs, including chromosomal location and relation to CpG islands and gene regions. Sheet b: SNP-CpG pairs where SNP is located in either C or G of the CpG site, so called CpG-SNPs. Sheet c: Additional annotation data for SNPs present in sheet a, based on HumanOmniExpress-12v1_J_Gene_Annotation_build37 (Illumina). Sheet d: Additional annotation data for CpGs present in sheet a, based on Infinium HumanMethylation 450 BeadChip [25] and probe cross-reactivity info as reported by Chen et al [88].
(XLSX)

S19 Table *trans*-mQTLs identified without BMI as a covariate. Sheet a: Identified *trans*-mQTL SNP-CpG pairs, including statistical results of associations and chromosomal location and relation to CpG islands and gene regions. Sheet b: Additional annotation data for SNPs present in sheet a, based on HumanOmniExpress-12v1_J_Gene_Annotation_build37 (Illumina). Sheet c: Additional annotation data for CpGs present in sheet a, based on Infinium HumanMethylation 450 BeadChip [25] and probe cross-reactivity info as reported by Chen et al [88].
(XLSX)

S20 Table Association between DNA methylation and gene expression in human adipose tissue. (XLSX)

S21 Table Sample characteristics of 4 different cohorts included in the study. (XLSX)

## Abbreviations

mQTL: methylation quantitative trait locus
SNP: single nucleotide polymorphism
BMI: body mass index
CIT: causal inference test
HbA1c: hemoglobin A1c
HDL: high-density lipoprotein
LDL: low density lipoprotein
HOMA-IR: homeostasis model insulin resistance
HOMA-B: homeostasis model beta-cell function
CRP: C-reactive protein
GWAS: genome-wide association study
TSS: transcription start site
UTR: untranslated region
WHR: waist-hip ratio
LD: linkage disequilibrium
KEGG: Kyoto Encyclopaedia of Genes and Genomes

## References

[1] McCarthy MI. Genomics, Type 2 Diabetes, and Obesity. N Engl J Med. 2010;363: 2339–2350. 10.1056/NEJMra0906948 21142536

[2] Franks PW, Ling C. Epigenetics and obesity: the devil is in the details. BMC Med. 2010;8: 88 10.1186/1741-7015-8-88 21176136 PMC3019199

[3] Ling C, Groop L. Epigenetics: A Molecular Link Between Environmental Factors and Type 2. Diabetes. 2009;58: 2718–2725. 10.2337/db09-1003 19940235 PMC2780862

[4] Bird A. Perceptions of epigenetics. Nature. 2007;447: 396–398. 10.1038/nature05913 17522671

[5] Bird A. DNA methylation patterns and epigenetic memory. Genes Dev. 2002;16: 6–21. 10.1101/gad.947102 11782440

[6] Jones PA. Functions of DNA methylation: islands, start sites, gene bodies and beyond. Nat Rev Genet. 2012;13: 484–492. 10.1038/nrg3230 22641018

[7] Brøns C, Jacobsen S, Nilsson E, Rönn T, Jensen CB, Storgaard H, et al Deoxyribonucleic acid methylation and gene expression of PPARGC1A in human muscle is influenced by high-fat overfeeding in a birth-weight-dependent manner. J Clin Endocrinol Metab. 2010;95: 3048–3056. 10.1210/jc.2009-2413 20410232

[8] Jacobsen SC, Brøns C, Bork-Jensen J, Ribel-Madsen R, Yang B, Lara E, et al Effects of short-term high-fat overfeeding on genome-wide DNA methylation in the skeletal muscle of healthy young men. Diabetologia. 2012;55: 3341–3349. 10.1007/s00125-012-2717-8 22961225

[9] Nitert MD, Dayeh T, Volkov P, Elgzyri T, Hall E, Nilsson E, et al Impact of an exercise intervention on DNA methylation in skeletal muscle from first-degree relatives of patients with type 2 diabetes. Diabetes. 2012;61: 3322–3332. 10.2337/db11-1653 23028138 PMC3501844

[10] Rönn T, Volkov P, Davegårdh C, Dayeh T, Hall E, Olsson AH, et al A six months exercise intervention influences the genome-wide DNA methylation pattern in human adipose tissue. PLoS Genet. 2013;9: e1003572 10.1371/journal.pgen.1003572 23825961 PMC3694844

[11] Anway MD, Cupp AS, Uzumcu M, Skinner MK. Epigenetic transgenerational actions of endocrine disruptors and male fertility. Science. 2005;308: 1466–1469. 10.1126/science.1108190 15933200 PMC11423801

[12] Chong S, Whitelaw E. Epigenetic germline inheritance. Curr Opin Genet Dev. 2004;14: 692–696. 10.1016/j.gde.2004.09.001 15531166

[13] Kaminsky ZA, Tang T, Wang S-C, Ptak C, Oh GHT, Wong AHC, et al DNA methylation profiles in monozygotic and dizygotic twins. Nat Genet. 2009;41: 240–245. 10.1038/ng.286 19151718

[14] Ollikainen M, Smith KR, Joo EJ-H, Ng HK, Andronikos R, Novakovic B, et al DNA methylation analysis of multiple tissues from newborn twins reveals both genetic and intrauterine components to variation in the human neonatal epigenome. Hum Mol Genet. 2010;19: 4176–4188. 10.1093/hmg/ddq336 20699328

[15] Nilsson E, Jansson PA, Perfilyev A, Volkov P, Pedersen M, Svensson MK, et al Altered DNA methylation and differential expression of genes influencing metabolism and inflammation in adipose tissue from subjects with type 2 diabetes. Diabetes. 2014; 10.2337/db13-1459 24812430

[16] Grundberg E, Meduri E, Sandling JK, Hedman AK, Keildson S, Buil A, et al Global analysis of DNA methylation variation in adipose tissue from twins reveals links to disease-associated variants in distal regulatory elements. Am J Hum Genet. 2013;93: 876–890. 10.1016/j.ajhg.2013.10.004 24183450 PMC3824131

[17] Zhang D, Cheng L, Badner JA, Chen C, Chen Q, Luo W, et al Genetic control of individual differences in gene-specific methylation in human brain. Am J Hum Genet. 2010;86: 411–419. 10.1016/j.ajhg.2010.02.005 20215007 PMC2833385

[18] Bell JT, Pai AA, Pickrell JK, Gaffney DJ, Pique-Regi R, Degner JF, et al DNA methylation patterns associate with genetic and gene expression variation in HapMap cell lines. Genome Biol. 2011;12: R10 10.1186/gb-2011-12-1-r10 21251332 PMC3091299

[19] Gibbs JR, van der Brug MP, Hernandez DG, Traynor BJ, Nalls MA, Lai S-L, et al Abundant Quantitative Trait Loci Exist for DNA Methylation and Gene Expression in Human Brain. PLoS Genet. 2010;6: e1000952 10.1371/journal.pgen.1000952 20485568 PMC2869317

[20] Olsson AH, Volkov P, Bacos K, Dayeh T, Hall E, Nilsson EA, et al Genome-Wide Associations between Genetic and Epigenetic Variation Influence mRNA Expression and Insulin Secretion in Human Pancreatic Islets. PLoS Genet. 2014;10: e1004735 10.1371/journal.pgen.1004735 25375650 PMC4222689

[21] Millstein J, Zhang B, Zhu J, Schadt EE. Disentangling molecular relationships with a causal inference test. BMC Genet. 2009;10: 23 10.1186/1471-2156-10-23 19473544 PMC3224661

[22] Dayeh TA, Olsson AH, Volkov P, Almgren P, Rönn T, Ling C. Identification of CpG-SNPs associated with type 2 diabetes and differential DNA methylation in human pancreatic islets. Diabetologia. 2013;56: 1036–1046. 10.1007/s00125-012-2815-7 23462794 PMC3622750

[23] Pociot F, McDermott MF. Genetics of type 1 diabetes mellitus. Genes Immun. 2002;3: 235–249. 10.1038/sj.gene.6363875 12140742

[24] Shiina T, Inoko H, Kulski JK. An update of the HLA genomic region, locus information and disease associations: 2004. Tissue Antigens. 2004;64: 631–649. 10.1111/j.1399-0039.2004.00327.x 15546336

[25] Bibikova M, Barnes B, Tsan C, Ho V, Klotzle B, Le JM, et al High density DNA methylation array with single CpG site resolution. Genomics. 2011;98: 288–295. 10.1016/j.ygeno.2011.07.007 21839163

[26] Wang J, Duncan D, Shi Z, Zhang B. WEB-based GEne SeT AnaLysis Toolkit (WebGestalt): update 2013. Nucleic Acids Res. 2013;41: W77–83. 10.1093/nar/gkt439 23703215 PMC3692109

[27] Hindorff, L.A., MacArthur, J., Morales, J., Junkins H.A., Hall, P.N., Klemm, A.K., & Manolio, T.A. A Catalog of Published Genome-Wide Association Studies. Available at: www.genome.gov/gwastudies. Accessed September 23 2013.

[28] Farooqi S, O’Rahilly S. Genetics of obesity in humans. Endocr Rev. 2006;27: 710–718. 10.1210/er.2006-0040 17122358

[29] Speliotes EK, Willer CJ, Berndt SI, Monda KL, Thorleifsson G, Jackson AU, et al Association analyses of 249,796 individuals reveal 18 new loci associated with body mass index. Nat Genet. 2010;42: 937–948. 10.1038/ng.686 20935630 PMC3014648

[30] Comuzzie AG, Cole SA, Laston SL, Voruganti VS, Haack K, Gibbs RA, et al Novel genetic loci identified for the pathophysiology of childhood obesity in the Hispanic population. PloS One. 2012;7: e51954 10.1371/journal.pone.0051954 23251661 PMC3522587

[31] Diabetes Genetics Initiative of Broad Institute of Harvard and MIT, Lund University, and Novartis Institutes of BioMedical Research, Saxena R, Voight BF, Lyssenko V, Burtt NP, de Bakker PIW, et al Genome-wide association analysis identifies loci for type 2 diabetes and triglyceride levels. Science. 2007;316: 1331–1336. 10.1126/science.1142358 17463246

[32] Lowe JK, Maller JB, Pe’er I, Neale BM, Salit J, Kenny EE, et al Genome-wide association studies in an isolated founder population from the Pacific Island of Kosrae. PLoS Genet. 2009;5: e1000365 10.1371/journal.pgen.1000365 19197348 PMC2628735

[33] Chambers JC, Elliott P, Zabaneh D, Zhang W, Li Y, Froguel P, et al Common genetic variation near MC4R is associated with waist circumference and insulin resistance. Nat Genet. 2008;40: 716–718. 10.1038/ng.156 18454146

[34] Sabatti C, Service SK, Hartikainen A-L, Pouta A, Ripatti S, Brodsky J, et al Genome-wide association analysis of metabolic traits in a birth cohort from a founder population. Nat Genet. 2009;41: 35–46. 10.1038/ng.271 19060910 PMC2687077

[35] Ryckman KK, Smith CJ, Jelliffe-Pawlowski LL, Momany AM, Berberich SL, Murray JC. Metabolic heritability at birth: implications for chronic disease research. Hum Genet. 2014;133: 1049–1057. 10.1007/s00439-014-1450-4 24850141 PMC4102629

[36] Manning AK, Hivert M-F, Scott RA, Grimsby JL, Bouatia-Naji N, Chen H, et al A genome-wide approach accounting for body mass index identifies genetic variants influencing fasting glycemic traits and insulin resistance. Nat Genet. 2012;44: 659–669. 10.1038/ng.2274 22581228 PMC3613127

[37] Gilad Y, Rifkin SA, Pritchard JK. Revealing the architecture of gene regulation: the promise of eQTL studies. Trends Genet TIG. 2008;24: 408–415. 10.1016/j.tig.2008.06.001 18597885 PMC2583071

[38] Zhu AZX, Renner CC, Hatsukami DK, Benowitz NL, Tyndale RF. CHRNA5-A3-B4 genetic variants alter nicotine intake and interact with tobacco use to influence body weight in Alaska Native tobacco users. Addict Abingdon Engl. 2013;108: 1818–1828. 10.1111/add.12250 PMC3775934 23692359

[39] Mahajan A, Sim X, Ng HJ, Manning A, Rivas MA, Highland HM, et al Identification and functional characterization of G6PC2 coding variants influencing glycemic traits define an effector transcript at the G6PC2-ABCB11 locus. PLoS Genet. 2015;11: e1004876 10.1371/journal.pgen.1004876 25625282 PMC4307976

[40] Williams MJ, Almén MS, Fredriksson R, Schiöth HB. What model organisms and interactomics can reveal about the genetics of human obesity. Cell Mol Life Sci CMLS. 2012;69: 3819–3834. 10.1007/s00018-012-1022-5 22618246 PMC11114734

[41] Fox CS, Liu Y, White CC, Feitosa M, Smith AV, Heard-Costa N, et al Genome-wide association for abdominal subcutaneous and visceral adipose reveals a novel locus for visceral fat in women. PLoS Genet. 2012;8: e1002695 10.1371/journal.pgen.1002695 22589738 PMC3349734

[42] Mackay DJG, Callaway JLA, Marks SM, White HE, Acerini CL, Boonen SE, et al Hypomethylation of multiple imprinted loci in individuals with transient neonatal diabetes is associated with mutations in ZFP57. Nat Genet. 2008;40: 949–951. 10.1038/ng.187 18622393

[43] Heid IM, Jackson AU, Randall JC, Winkler TW, Qi L, Steinthorsdottir V, et al Meta-analysis identifies 13 new loci associated with waist-hip ratio and reveals sexual dimorphism in the genetic basis of fat distribution. Nat Genet. 2010;42: 949–960. 10.1038/ng.685 20935629 PMC3000924

[44] Yang J, Loos RJF, Powell JE, Medland SE, Speliotes EK, Chasman DI, et al FTO genotype is associated with phenotypic variability of body mass index. Nature. 2012;490: 267–272. 10.1038/nature11401 22982992 PMC3564953

[45] Dupuis J, Langenberg C, Prokopenko I, Saxena R, Soranzo N, Jackson AU, et al New genetic loci implicated in fasting glucose homeostasis and their impact on type 2 diabetes risk. Nat Genet. 2010;42: 105–116. 10.1038/ng.520 20081858 PMC3018764

[46] Soranzo N, Sanna S, Wheeler E, Gieger C, Radke D, Dupuis J, et al Common Variants at 10 Genomic Loci Influence Hemoglobin A1C Levels via Glycemic and Nonglycemic Pathways. Diabetes. 2010;59: 3229–3239. 10.2337/db10-0502 20858683 PMC2992787

[47] Willer CJ, Schmidt EM, Sengupta S, Peloso GM, Gustafsson S, Kanoni S, et al Discovery and refinement of loci associated with lipid levels. Nat Genet. 2013;45: 1274–1283. 10.1038/ng.2797 24097068 PMC3838666

[48] Rönn T, Volkov P, Gillberg L, Kokosar M, Perfilyev A, Jacobsen AL, et al Impact of age, BMI and HbA1c levels on the genome-wide DNA methylation and mRNA expression patterns in human adipose tissue and identification of epigenetic biomarkers in blood. Hum Mol Genet. 2015;24: 3792–3813. 10.1093/hmg/ddv124 25861810

[49] Groop L, Pociot F. Genetics of diabetes—are we missing the genes or the disease? Mol Cell Endocrinol. 2014;382: 726–739. 10.1016/j.mce.2013.04.002 23587769

[50] Steinthorsdottir V, Thorleifsson G, Reynisdottir I, Benediktsson R, Jonsdottir T, Walters GB, et al A variant in CDKAL1 influences insulin response and risk of type 2 diabetes. Nat Genet. 2007;39: 770–775. 10.1038/ng2043 17460697

[51] Frayling TM, Timpson NJ, Weedon MN, Zeggini E, Freathy RM, Lindgren CM, et al A common variant in the FTO gene is associated with body mass index and predisposes to childhood and adult obesity. Science. 2007;316: 889–894. 10.1126/science.1141634 17434869 PMC2646098

[52] Kilpeläinen TO, Zillikens MC, Stančákova A, Finucane FM, Ried JS, Langenberg C, et al Genetic variation near IRS1 associates with reduced adiposity and an impaired metabolic profile. Nat Genet. 2011;43: 753–760. 10.1038/ng.866 21706003 PMC3262230

[53] Gluckman PD, Hanson MA, Buklijas T, Low FM, Beedle AS. Epigenetic mechanisms that underpin metabolic and cardiovascular diseases. Nat Rev Endocrinol. 2009;5: 401–408. 10.1038/nrendo.2009.102 19488075

[54] Ling C, Del Guerra S, Lupi R, Rönn T, Granhall C, Luthman H, et al Epigenetic regulation of PPARGC1A in human type 2 diabetic islets and effect on insulin secretion. Diabetologia. 2008;51: 615–622. 10.1007/s00125-007-0916-5 18270681 PMC2270364

[55] Yang BT, Dayeh TA, Kirkpatrick CL, Taneera J, Kumar R, Groop L, et al Insulin promoter DNA methylation correlates negatively with insulin gene expression and positively with HbA(1c) levels in human pancreatic islets. Diabetologia. 2011;54: 360–367. 10.1007/s00125-010-1967-6 21104225 PMC3017313

[56] Yang BT, Dayeh TA, Volkov PA, Kirkpatrick CL, Malmgren S, Jing X, et al Increased DNA methylation and decreased expression of PDX-1 in pancreatic islets from patients with type 2 diabetes. Mol Endocrinol Baltim Md. 2012;26: 1203–1212. 10.1210/me.2012-1004 PMC5416998 22570331

[57] Barrès R, Osler ME, Yan J, Rune A, Fritz T, Caidahl K, et al Non-CpG methylation of the PGC-1alpha promoter through DNMT3B controls mitochondrial density. Cell Metab. 2009;10: 189–198. 10.1016/j.cmet.2009.07.011 19723495

[58] Dick KJ, Nelson CP, Tsaprouni L, Sandling JK, Aïssi D, Wahl S, et al DNA methylation and body-mass index: a genome-wide analysis. Lancet. 2014;383: 1990–1998. 10.1016/S0140-6736(13)62674-4 24630777

[59] Dayeh T, Volkov P, Salö S, Hall E, Nilsson E, Olsson AH, et al Genome-wide DNA methylation analysis of human pancreatic islets from type 2 diabetic and non-diabetic donors identifies candidate genes that influence insulin secretion. PLoS Genet. 2014;10: e1004160 10.1371/journal.pgen.1004160 24603685 PMC3945174

[60] Ling C, Poulsen P, Simonsson S, Rönn T, Holmkvist J, Almgren P, et al Genetic and epigenetic factors are associated with expression of respiratory chain component NDUFB6 in human skeletal muscle. J Clin Invest. 2007;117: 3427–3435. 10.1172/JCI30938 17948130 PMC2030455

[61] Drong AW, Nicholson G, Hedman ÅK, Meduri E, Grundberg E, Small KS, et al The Presence of Methylation Quantitative Trait Loci Indicates a Direct Genetic Influence on the Level of DNA Methylation in Adipose Tissue. PLoS ONE. 2013;8: e55923 10.1371/journal.pone.0055923 23431366 PMC3576415

[62] Eckhardt F, Lewin J, Cortese R, Rakyan VK, Attwood J, Burger M, et al DNA methylation profiling of human chromosomes 6, 20 and 22. Nat Genet. 2006;38: 1378–1385. 10.1038/ng1909 17072317 PMC3082778

[63] Irizarry RA, Ladd-Acosta C, Wen B, Wu Z, Montano C, Onyango P, et al The human colon cancer methylome shows similar hypo- and hypermethylation at conserved tissue-specific CpG island shores. Nat Genet. 2009;41: 178–186. 10.1038/ng.298 19151715 PMC2729128

[64] Ziller MJ, Gu H, Müller F, Donaghey J, Tsai LT-Y, Kohlbacher O, et al Charting a dynamic DNA methylation landscape of the human genome. Nature. 2013;advance online publication. 10.1038/nature12433 PMC3821869 23925113

[65] Dehghan A, Dupuis J, Barbalic M, Bis JC, Eiriksdottir G, Lu C, et al Meta-analysis of genome-wide association studies in >80 000 subjects identifies multiple loci for C-reactive protein levels. Circulation. 2011;123: 731–738. 10.1161/CIRCULATIONAHA.110.948570 21300955 PMC3147232

[66] Voight BF, Scott LJ, Steinthorsdottir V, Morris AP, Dina C, Welch RP, et al Twelve type 2 diabetes susceptibility loci identified through large-scale association analysis. Nat Genet. 2010;42: 579–589. 10.1038/ng.609 20581827 PMC3080658

[67] Speliotes EK, Willer CJ, Berndt SI, Monda KL, Thorleifsson G, Jackson AU, et al Association analyses of 249,796 individuals reveal 18 new loci associated with body mass index. Nat Genet. 2010;42: 937–948. 10.1038/ng.686 20935630 PMC3014648

[68] Scott RA, Lagou V, Welch RP, Wheeler E, Montasser ME, Luan J, et al Large-scale association analyses identify new loci influencing glycemic traits and provide insight into the underlying biological pathways. Nat Genet. 2012;44: 991–1005. 10.1038/ng.2385 22885924 PMC3433394

[69] Feinberg AP, Irizarry RA. Stochastic epigenetic variation as a driving force of development, evolutionary adaptation, and disease. Proc Natl Acad Sci. 2009; 200906183. doi: 10.1073/pnas.0906183107 PMC2868296 20080672

[70] Liu Y, Aryee MJ, Padyukov L, Fallin MD, Hesselberg E, Runarsson A, et al Epigenome-wide association data implicate DNA methylation as an intermediary of genetic risk in rheumatoid arthritis. Nat Biotechnol. 2013;31: 142–147. 10.1038/nbt.2487 23334450 PMC3598632

[71] Ma L, Yang J, Runesha HB, Tanaka T, Ferrucci L, Bandinelli S, et al Genome-wide association analysis of total cholesterol and high-density lipoprotein cholesterol levels using the Framingham Heart Study data. BMC Med Genet. 2010;11: 55 10.1186/1471-2350-11-55 20370913 PMC2867786

[72] Cheung YH, Watkinson J, Anastassiou D. Conditional meta-analysis stratifying on detailed HLA genotypes identifies a novel type 1 diabetes locus around TCF19 in the MHC. Hum Genet. 2011;129: 161–176. 10.1007/s00439-010-0908-2 21076979 PMC3020293

[73] Zeggini E, Scott LJ, Saxena R, Voight BF, Marchini JL, Hu T, et al Meta-analysis of genome-wide association data and large-scale replication identifies additional susceptibility loci for type 2 diabetes. Nat Genet. 2008;40: 638–645. 10.1038/ng.120 18372903 PMC2672416

[74] Das SK, Sharma NK. Expression quantitative trait analyses to identify causal genetic variants for type 2 diabetes susceptibility. World J Diabetes. 2014;5: 97–114. 10.4239/wjd.v5.i2.97 24748924 PMC3990322

[75] Elgzyri T, Parikh H, Zhou Y, Dekker Nitert M, Rönn T, Segerström ÅB, et al First-degree relatives of type 2 diabetic patients have reduced expression of genes involved in fatty acid metabolism in skeletal muscle. J Clin Endocrinol Metab. 2012;97: E1332–1337. 10.1210/jc.2011-3037 22547424

[76] Brøns C, Jensen CB, Storgaard H, Alibegovic A, Jacobsen S, Nilsson E, et al Mitochondrial function in skeletal muscle is normal and unrelated to insulin action in young men born with low birth weight. J Clin Endocrinol Metab. 2008;93: 3885–3892. 10.1210/jc.2008-0630 18628517

[77] Jørgensen SW, Brøns C, Bluck L, Hjort L, Færch K, Thankamony A, et al Metabolic response to 36 hours of fasting in young men born small vs appropriate for gestational age. Diabetologia. 2014; 10.1007/s00125-014-3406-6 25287712

[78] Purcell S, Neale B, Todd-Brown K, Thomas L, Ferreira MAR, Bender D, et al PLINK: a tool set for whole-genome association and population-based linkage analyses. Am J Hum Genet. 2007;81: 559–575. 10.1086/519795 17701901 PMC1950838

[79] Gentleman RC, Carey VJ, Bates DM, Bolstad B, Dettling M, Dudoit S, et al Bioconductor: open software development for computational biology and bioinformatics. Genome Biol. 2004;5: R80 10.1186/gb-2004-5-10-r80 15461798 PMC545600

[80] Du P, Zhang X, Huang C-C, Jafari N, Kibbe WA, Hou L, et al Comparison of Beta-value and M-value methods for quantifying methylation levels by microarray analysis. BMC Bioinformatics. 2010;11: 587 10.1186/1471-2105-11-587 21118553 PMC3012676

[81] Du P, Kibbe WA, Lin SM. lumi: a pipeline for processing Illumina microarray. Bioinforma Oxf Engl. 2008;24: 1547–1548. 10.1093/bioinformatics/btn224 18467348

[82] Johnson WE, Li C, Rabinovic A. Adjusting batch effects in microarray expression data using empirical Bayes methods. Biostatistics. 2007;8: 118–127. 10.1093/biostatistics/kxj037 16632515

[83] Carvalho BS, Irizarry RA. A framework for oligonucleotide microarray preprocessing. Bioinforma Oxf Engl. 2010;26: 2363–2367. 10.1093/bioinformatics/btq431 PMC2944196 20688976

[84] Shabalin AA. Matrix eQTL: ultra fast eQTL analysis via large matrix operations. Bioinformatics. 2012;28: 1353–1358. 10.1093/bioinformatics/bts163 22492648 PMC3348564

[85] Holger Schwender, Qing Li, Christoph Neumann, Margaret Taub, Ingo Ruczinski. trio: Testing of SNPs and SNP Interactions in Case-Parent Trio Studies. R package version 3.0.0. 2013;

[86] Johnson AD, Handsaker RE, Pulit SL, Nizzari MM, O’Donnell CJ, Bakker PIW de. SNAP: a web-based tool for identification and annotation of proxy SNPs using HapMap. Bioinformatics. 2008;24: 2938–2939. 10.1093/bioinformatics/btn564 18974171 PMC2720775

[87] R Core Team. R: A language and environment for statistical computing. R Foundation for Statistical Computing, Vienna, Austria. 2013;

[88] Chen Y, Lemire M, Choufani S, Butcher DT, Grafodatskaya D, Zanke BW, et al Discovery of cross-reactive probes and polymorphic CpGs in the Illumina Infinium HumanMethylation450 microarray. Epigenetics. 2013;8: 203–209. 10.4161/epi.23470 23314698 PMC3592906

